# Machine Learning Predicts the Yeast Metabolome from the Quantitative Proteome of Kinase Knockouts

**DOI:** 10.1016/j.cels.2018.08.001

**Published:** 2018-09-26

**Authors:** Aleksej Zelezniak, Jakob Vowinckel, Floriana Capuano, Christoph B. Messner, Vadim Demichev, Nicole Polowsky, Michael Mülleder, Stephan Kamrad, Bernd Klaus, Markus A. Keller, Markus Ralser

**Affiliations:** 1 The Francis Crick Institute, Molecular Biology of Metabolism laboratory, London, UK; 2 Department of Biochemistry and Cambridge Systems Biology Centre, University of Cambridge, Cambridge, UK; 3 Department of Biology and Biological Engineering, Chalmers University of Technology, Gothenburg, Sweden; 4 Science for Life Laboratory, KTH – Royal Institute of Technology, Stockholm, Sweden; 5 Biognosys AG, Schlieren, Switzerland; 6 Centre for Statistical Data Analysis, European Molecular Biology Laboratory (EMBL), Heidelberg, Germany; 7 Department of Genetics, Evolution and Environment, University College London, London, UK; 8 Medical University of Innsbruck, Innsbruck, Austria; 9 Department of Biochemistry, Charité Universitaetsmedizin Berlin, Berlin, Germany

**Keywords:** metabolism, high-throughput proteomics, hierarchical regulation, enzyme abundance, metabolic control analysis, machine learning, genotype-phenotype problem, multi-omics

## Abstract

A challenge in solving the genotype-to-phenotype relationship is to predict a cell’s metabolome, believed to correlate poorly with gene expression. Using comparative quantitative proteomics, we found that differential protein expression in 97 *Saccharomyces cerevisiae* kinase deletion strains is non-redundant and dominated by abundance changes in metabolic enzymes. Associating differential enzyme expression landscapes to corresponding metabolomes using network models provided reasoning for poor proteome-metabolome correlations; differential protein expression redistributes flux control between many enzymes acting in concert, a mechanism not captured by one-to-one correlation statistics. Mapping these regulatory patterns using machine learning enabled the prediction of metabolite concentrations, as well as identification of candidate genes important for the regulation of metabolism. Overall, our study reveals that a large part of metabolism regulation is explained through coordinated enzyme expression changes. Our quantitative data indicate that this mechanism explains more than half of metabolism regulation and underlies the interdependency between enzyme levels and metabolism, which renders the metabolome a predictable phenotype.

## Highlights

• The proteome of kinase knockouts is dominated by enzyme abundance changes
• The enzyme expression profiles of kinase knockouts are non-redundant
• Metabolism is regulated by many expression changes acting in concert
• Machine learning accurately predicts the metabolome from enzyme abundance

## Graphical Abstract

**Figure:**
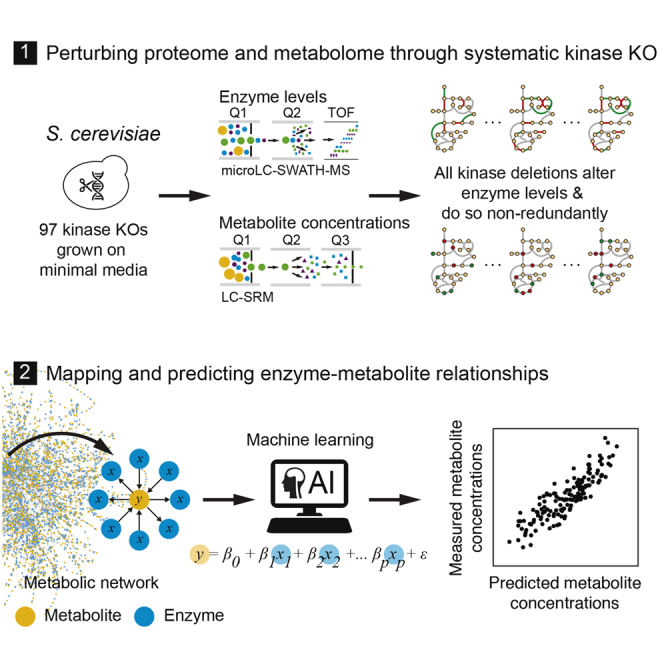

## Teaser

Predicting metabolomes from gene expression data is a key challenge in understanding the genotype-phenotype relationship. Studying the enzyme expression proteome in kinase knockouts, we reveal the importance of a so far overlooked metabolism-regulatory mechanism. Enzyme expression changes are impacting on metabolite levels through many changes acting in concert. We show that one can map regulatory enzyme expression patterns using machine learning and use them to predict the metabolome of kinase-deficient cells on the basis of their enzyme expression proteome. Our study quantifies the role of enzyme abundance in the regulation of metabolism and by doing so reveals the potential of machine learning in gaining understanding about complex metabolism regulation.

## Introduction

Despite the fact that metabolism is intensively studied, one still debates about how much of metabolic regulation is explained by metabolic self-regulation and by regulation of enzyme activity and how much is dependent on enzyme abundance changes. The current literature is split, in essence, between two seemingly contrasting observations. On the one hand, available quantitative models can explain only a minor fraction of metabolite concentrations on the basis of gene expression data (Fendt et al., 2010, Kresnowati et al., 2006, Zelezniak et al., 2014). Moreover, metabolite concentrations seem to correlate much better with metabolic fluxes than with enzyme expression levels (Chubukov et al., 2013, Hackett et al., 2016, Millard et al., 2017). These results seem to suggest that the post-translational regulation, metabolic self-regulation, and allostery are dominant in metabolism regulation.

On the other hand, however, large fractions of the transcriptome respond to changes in metabolism (Alam et al., 2016, Bradley et al., 2009, Chechik et al., 2008, Kresnowati et al., 2006, Murray et al., 2007, Tu et al., 2005, Urbanczyk-Wochniak et al., 2003). The expression changes are centered on metabolites that change in concentration (Patil and Nielsen, 2005, Zelezniak et al., 2010), while systematically recorded transcriptomes and proteomes of metabolically perturbed yeast correlate with metabolic flux distributions (Alam et al., 2016). Hence, despite poor correlation values between individual enzyme levels and metabolism, changes in metabolism seem tightly intertwined with gene expression changes. Indeed, all metabolism-regulating transcriptional and signaling networks identified to date, such as AMP-activated protein kinase (AMPK) (Mihaylova and Shaw, 2011), mechamTOR (González and Hall, 2017), or GCN2/4 (Zaborske et al., 2010), trigger metabolic gene expression changes.

A potential explanation for this apparent paradox could be provided by the nature of enzyme-metabolite relationships. Reaction mechanisms (Braakman and Smith, 2013), the self-regulatory nature of metabolic networks (Alam et al., 2017, Chubukov et al., 2013, Hackett et al., 2016, Millard et al., 2017), post-translational regulation (Daran-Lapujade et al., 2007, Gonçalves et al., 2017, Nilsson et al., 2017, Oliveira et al., 2012), and the topological organization of metabolism that routes evolutionarily in the underlying chemistry (Burgard et al., 2004, Keller et al., 2015, Zelezniak et al., 2014) all dictate that the relationship between enzyme function and metabolites is both multifactorial and dynamic.

We selected a genome-spanning collection of 97 kinase gene deletion (“knockout”) *Saccharomyces cerevisiae* strains, known to exhibit differences in metabolism (Bodenmiller et al., 2010, Schulz et al., 2014, van Wageningen et al., 2010, Winzeler et al., 1999), noting that the gene expression changes in these strains remained uninvestigated in the context of their metabolome. A recently developed high-throughput proteomic platform (Vowinckel et al., 2018) was used to quantify enzyme expression and link enzyme expression changes to metabolite concentrations measured.

All kinase deletions triggered enzyme expression changes. Moreover, enzyme abundance changes dominated quantitatively over other differentially expressed functional protein categories in the kinase knockout proteomes. Using metabolic control analysis (MCA), we then revealed the importance of largely overlooked mechanisms in metabolic regulation. The proteomic changes detected were so broad that metabolic control shifts between different sets of enzymes. As a consequence, metabolic regulation becomes sensitive to global changes in gene expression, rather than being correlated to individual enzymes. To capture the multifactorial relationships, we developed a data-driven framework based on machine learning (ML). Training the algorithms on the basis of the metabolic network topology, we achieved the quantitative prediction of entire cellular metabolomes, thereby quantifying the role of enzyme abundance changes in metabolism regulation.

## Results

### Kinase Knockout Proteomes Are Dominated by Differential Enzyme Expression

**Figure 1:**
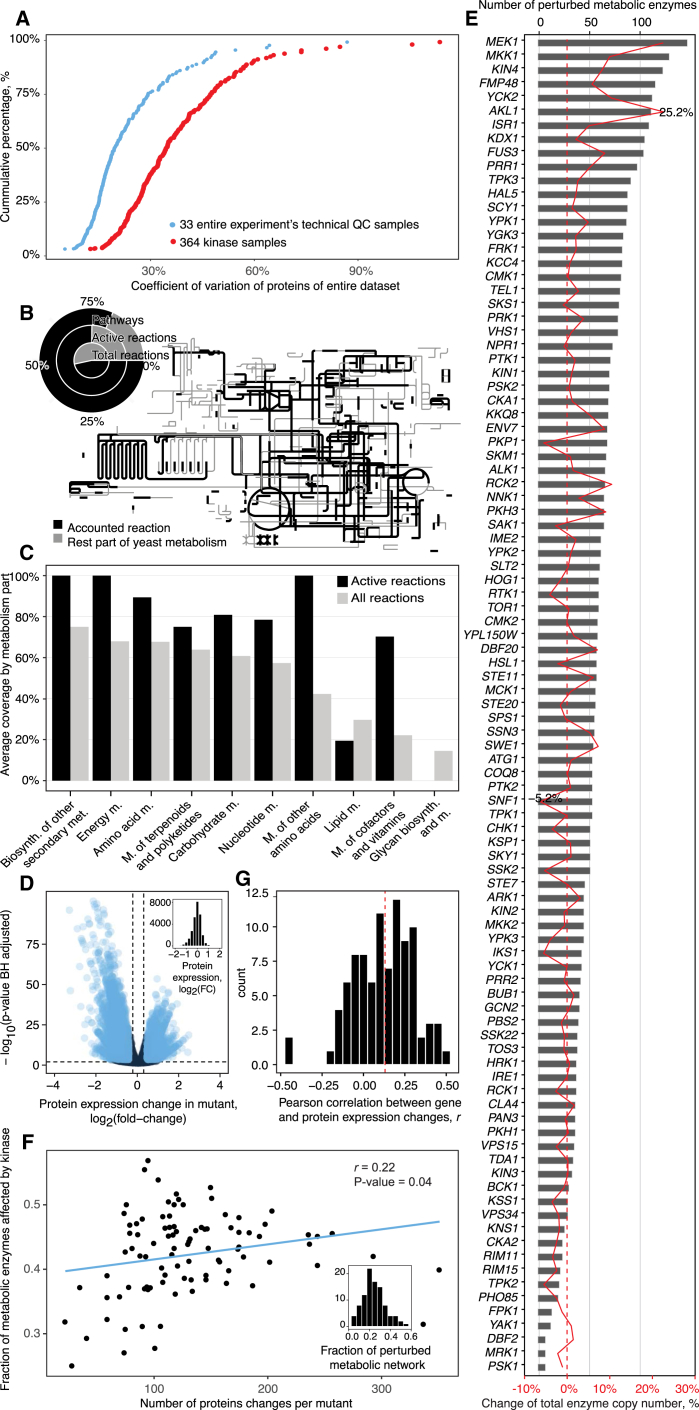
A Deletion of Each of the 97 Non-essential Yeast Protein Kinases Triggers Broad and Quantitatively Strong Changes in Metabolic Enzyme Expression (A) Biological versus technical variability in a large-scale proteomic experiment. The coefficient of variation (CV) of enzymes at whole-process technical and biological levels. Cyan dots indicate CVs of a standardized proteome digest (quality control [QC] sample) that was used to monitor instrument performance over a 4-month acquisition period. QCs were used to normalize for batch effects, as well as to determine adequate cutoff values for determining differential protein expression. See also Figure S2 and STAR Methods. (B) Projection of quantified enzymes on the KEGG metabolic pathway map using iPath (Yamada et al., 2011) illustrates a connected network coverage, indicating comprehensive coverage of the active metabolic reactions by the proteome data. The black lines represent reactions catalyzed by at least one quantified enzyme; gray lines represent enzymatic reactions for which no enzyme was quantified. Circle plot: obtained coverage in comparison to all metabolic pathways’ theoretically active reactions (reactions that couple to biomass growth) in yeast as determined by flux-coupling analysis (Burgard et al., 2004) and compared to all KEGG-annotated reactions of the yeast metabolic network. (C) MicroLC-SWATH-MS proteomes capture large parts of the active enzymome. The representation of KEGG metabolic pathways by enzymes quantified in each proteome, shown as average coverage of metabolic pathways per KEGG metabolism category (KEGG BRITE hierarchy level B). A reaction was considered covered if >1 enzyme with the corresponding EC number was quantified. (D) Each of the 97 kinase deletions affects enzyme expression levels (volcano plot). Differential enzyme expression in all mutants is compared to the parental strain. Cutoffs were determined using repeated measurements on the control sample (STAR Methods) and determined as a fold change cutoff > |log_2_(1.4/0.714)|, Benjamini-Hochberg (Benjamini and Hochberg, 1995) adjusted p < 0.01, cyan colors indicate differentially expressed enzymes. Inset: the distribution of fold change values between mutants and parental strains. (E) The total number of metabolic enzymes affected by kinase deletions illustrated for each kinase. Red line: influence of the individual kinase deletion in relation to the total enzyme copy number in percent. Copy number changes were obtained by calibrating the proteome data according to the absolute values of protein expression (Kulak et al., 2014) (Details are given in the STAR Methods section). (F) Enzyme abundance changes account for a major fraction of all differentially expressed proteins as quantified in the kinase knockouts, and the relative contribution of enzymes has a low correlation with the total size of the proteomic perturbation. The y axis represents the fraction of the differentially expressed metabolic enzymes out of all quantified proteins. Inset: kinase deletions affect up to 49% of all quantified enzymes as denoted by the total of the metabolic network, summing up in all strains to 39% of the measured impact of the total kinome on protein expression. (G) Correlation of metabolic enzymes between proteome and transcriptomes (van Wageningen et al., 2010) expressed as fold changes. See also Figure S5.

*S. cerevisiae* kinase gene knockout strains (Winzeler et al., 1999) were rendered prototrophic by introducing the pHLUM minichromosome (Mülleder et al., 2012) and cultivated in the absence of amino acid supplementation (STAR Methods; Figure S1 for growth rates). The measurement of the 97 proteomes mounted to 397 whole-proteome samples (triplicates plus controls) processed using the data-independent acquisition method SWATH-MS (Gillet et al., 2012) and the workflow optimized for achieving high quantification precision at large sample numbers (Vowinckel et al., 2018). The median coefficient of variation of protein abundance obtained was 19% (Figures 1A and S2). Cutoff values for differential protein expression were determined experimentally and defined as a 40% change, and we used a Benjamini-Hochberg (BH) adjusted p value cutoff of 0.01 (STAR Methods). To confirm that differentially expressed genes were specific to kinase deletions, a subset of 10 strains was mated to a wild-type (WT) strain or to a complementary kinase-knockout; in all cases, the proteomes in which the kinases were reintroduced centered closer to the WT proteomes and were different from homozygous mutants (Figure S3).

To capture enzyme expression values, we processed all SWATH proteomes using a spectral library generated from a soluble yeast protein extract and obtained a matrix that connects the 97 kinase deletions to the abundance of 286 metabolic enzymes (median q value < 0.01, hereafter called the “kinase-metabolic enzyme matrix”). These represent over 75% of the metabolic reactions that are coupled to biomass growth (STAR Methods; Figure 1B) and capture cytoplasmic metabolism close to completion (Figure 1C).

Each proteome was characterized by strong differential enzyme expression (Figure 1D). By comparing the kinase-metabolic enzyme matrix of each knockout strain to the full SWATH proteomes, we observed that 39% of all detected protein expression changes were attributable to metabolic enzymes. On average, a kinase deletion affected the abundance of 56 metabolic enzymes; the minimum was 7 enzymes differentially expressed upon deletion of *DBF2*, and the maximum was 140 enzymes upon deletion of *MEK1* (Figure 1E). Expressed in absolute protein copy numbers, up to 25% of the total cell protein abundance is affected by kinase deletions acting on enzyme abundance (Figure 1E). It is unlikely that these changes reflect a common pleiotropic mechanism. For example, although yeast growth rate itself is understood to control gene expression (Gasch et al., 2000), less than 10% of the total proteome changes in our kinase knockout strains could be explained by changes in growth rate (Figure S4). Moreover, there was no strong correlation (*r* = 0.22, p = 0.04) between the total number of differentially expressed proteins and the fraction of differentially expressed metabolic enzyme genes (Figure 1F).

We then compared protein expression levels to microarray-based transcriptional profiles (van Wageningen et al., 2010). The transcriptional profiles correlated significantly with the enzyme expression proteomes (Figure 1G; STAR Methods; Figure S5). Most likely, as the strains in the microarray-study were cultivated in amino-acid-supplemented media, the absolute correlation values were lower than previous studies in which yeast cells are grown under the exact same condition (Alam et al., 2016, Lahtvee et al., 2017, Marguerat et al., 2012). The significant correlation nonetheless indicates that transcriptional regulation is implicated in the protein abundance changes as detected. This analysis further revealed that enzymes differentially expressed are enriched among the highly expressed genes, while in the low abundant fraction of the transcriptome, differential enzyme expression is also significant but less prevalent (Figure 1F; STAR Methods). In parallel, a weak but significant correlation was obtained between protein degradation rates (Christiano et al., 2014) and the likelihood of an enzyme to be differentially expressed (Figure S6). Kinase deletions hence affect enzyme abundance both via hierarchical regulation, as well as via mechanisms that affect protein turnover.

### Enzyme Expression Signatures Reveal a High Degree of Specificity in Kinase Function

In a few cases, we observed a significant overlap between the enzyme proteomes, which seems to suggest common biological function. For example, deletion of MAPK kinases *HOG1* and *KSS1*, which share upstream signaling components (Saito and Tatebayashi, 2004), caused enzyme proteomes that did overlap in 25% and 33% of up- and down-regulated enzymes, respectively. Moreover, kinases of the same protein family were significantly more likely to also affect similar enzyme targets (one-way ANOVA p = 0.0092). For instance, the Ca2+/calmodulin-dependent protein kinase (CamK) and Casein kinase I (CKI) families revealed significant co-regulation in enzyme abundances (p = 0.0091) (Figure S10).

**Figure 2:**
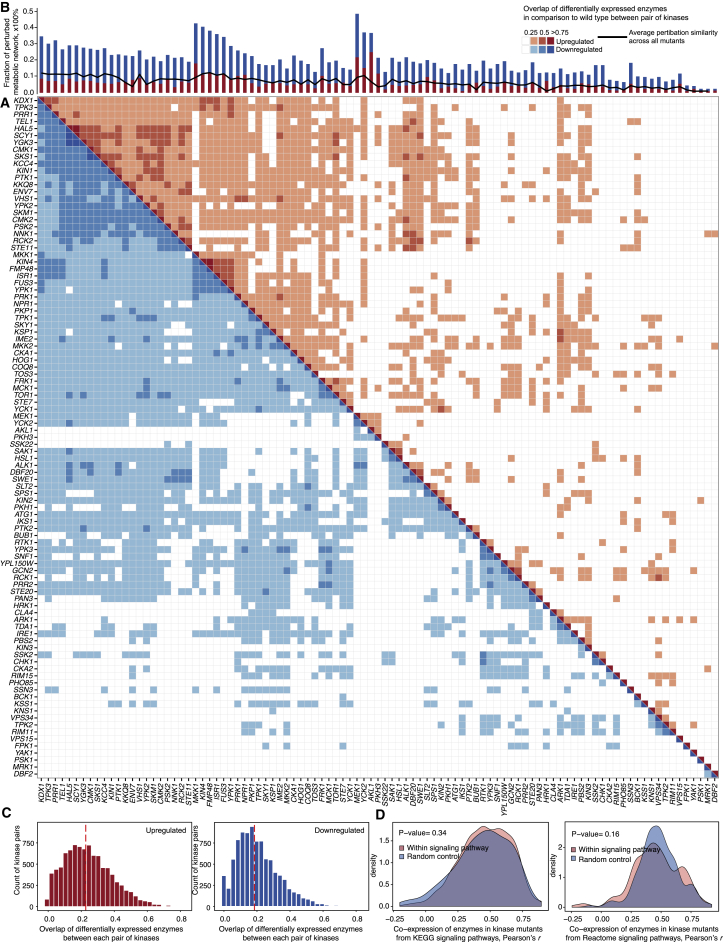
The Deletion of Each Yeast Kinase Triggers a Unique Reconfiguration of Enzyme Expression in the Cell (A) Similarity and overlap between enzyme expression proteomes obtained upon kinase deletion in *S. cerevisiae*. Each cell represents the overlap in the compendium of differentially expressed enzymes (relative to the parental strain BY4741-pHLUM) between any pair of kinase knockouts. An enzyme is considered differentially expressed if the fold change > |log_2_(1.4/0.714)|, BH adj. p < 0.01. The matrix distinguishes between upregulated (red, upper right part of the matrix) and downregulated (blue, lower left part) enzymes. For illustration purposes, rows and columns are clustered according to the Jaccard distance between the proteomes, disregarding the directionality of the expression changes. The overlap between each pair of proteomes is shown as Jaccard similarity. (B) The fraction of differentially expressed metabolic enzymes in comparison to total differential protein expression in all kinase mutants (bar chart). The absolute average similarity of kinase deletion enzyme proteomes, across all kinase mutants, is depicted as a black line. The typical kinase deletion causes a unique enzyme expression signature, with a median dissimilarity between kinase proteome pairs of 88% (average overlap between enzymes differentially expressed = 12%). (C) The typical overlap of perturbed enzyme proteomes in kinases mutants is not more than ∼25% (dotted median line). (D) Enzyme expression changes (log_2_-fold change) are not better explained by the signaling pathway annotations as obtained from KEGG or Reactome databases compared to randomly assembled pathways. More comparisons are provided in Figures S9 and S10.

For most kinase deletions, however, the precise proteome data revealed a high degree of specificity. Moreover, the proteomes suggest that enzyme expression regulation is too complex to be explained by linear signaling pathways. A Jaccard distance calculated between each kinase pair’s enzyme expression signature, as well as hierarchical clustering using complete linkage agglomeration (Figure 2A), revealed that 98% of kinase pairs have less than 50% overlap in differential enzyme expression. On average, two kinase enzyme proteomes overlap by less than 12% (Figure 2B). If expressed as a Pearson’s correlation, three-quarters of the proteome changes in the typical kinase deletion were specific (Figure S7). A sensitivity analysis ruled out a thresholding artifact; indeed, with more conservative thresholds, the specificity of kinase proteomes is revealed more robustly (Figure S7).

Consistently, the signaling pathway annotations as assembled in both KEGG and Reactome databases (Fabregat et al., 2016, Kanehisa et al., 2016) could not explain enzyme co-expression and indeed were not more predictive about enzyme co-expression as random networks (identical Pearson’s correlation coefficient, Wilcoxon rank-sum test, p > 0.05 [Figures 2D and S8]). This result was corroborated by comparing the overlaps of differentially expressed enzymes. A borderline significant association with the Reactome database pathways was explained because in the database, one pair of paralogous serine/threonine kinases (*YPK1* and *YPK2*, overlapping in 31% of their enzyme expression changes) is associated with 42% of signaling pathways (Wilcoxon rank-sum test, p = 0.03; Figure S9). When this pair is removed, no significant correlation of signaling pathway associations and enzyme co-expression was observed.

### Enzyme Expression Affects Steady-State Metabolite Pools

**Figure 3:**
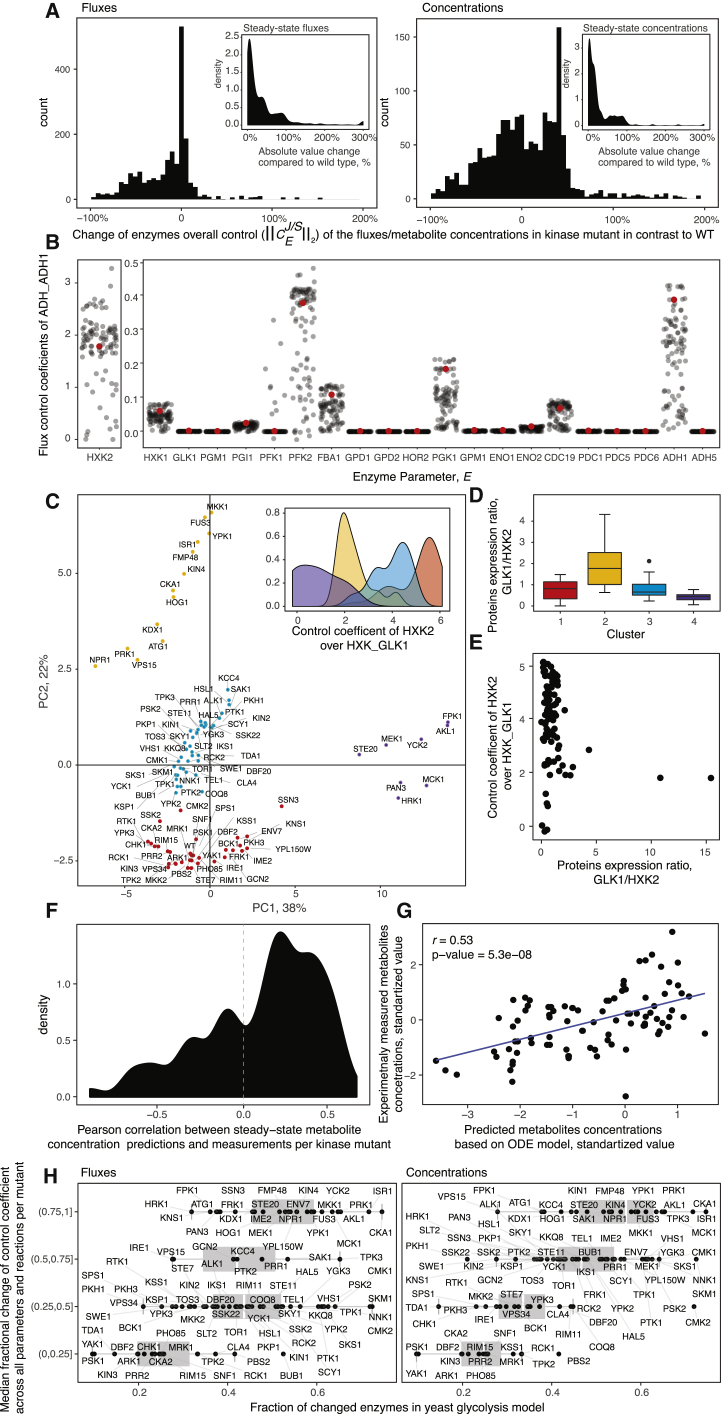
Enzyme Expression Affects Steady-State Metabolism through Redistributing Flux Control (A) Overall control coefficients of concentrations (CCC) and fluxes (FCC) are changed in kinase deletion strains comparing to WT due to the differential expression of multiple enzymes. The overall FCCs were calculated as described in Millard et al. (2017), i.e., taking for every enzyme the second norm over all its concentrations and FCCs that were parameterized on it (STAR Methods). Insets: simulated steady-state changes of fluxes and metabolite levels in kinase mutants in comparison to WT. (B) FCCs (*C*^*J*^*E*) over alcohol dehydrogenase (EC 1.1.1.1) reaction (y axis) by corresponding glycolytic enzymes (x axis) upon adjusting protein expression levels in a yeast glycolysis model as measured in each kinase knockout. Red dots indicate the WT strain values. To preserve the original scales, the control coefficients for *HXK2* are plotted on a separate y axis. Differential enzyme expression substantially redistributes control coefficients in multiple kinases to different enzymes. (C) Principal-component analysis (PCA) of FCCs for every kinase gene deletion mutant reveals a distinct set of expression patterns that influences control over glycolysis. FCCs are not scaled (See also Figure S12). Axes labels represent the percentage of total variance explained by each of the PCs. Colors represent established flux regulatory clusters (STAR Methods). Cluster separation is mainly driven (inset) by control of *HXK2* on *GLK1* reaction. (D) Within each flux regulatory cluster, large differences between the *GLK1/HXK2* expression ratio are observed. Corresponding p values for each pair of clusters using Wilcoxon rank-sum test (1 versus 2 p = 5.4e−05; 1 versus 3 p = 1.5e−02; 1 versus 4 p = 6.01e−01; 2 versus 3 p = 6.35e−05; 2 versus 4 p = 2.01e−03; 3 versus 4 p = 3.39e−01). (E) Flux control is a systemic property that depends on the coordinated expression of multiple enzymes. Even the most dominant single contributor (*GLK1/HXK2* ratio, [x axis]) alone cannot explain the variation of flux control coefficients (y axis) as a result of differential enzyme expression. (F) Measured metabolite concentrations correlate with steady-state predictions by the enzyme-level adjusted kinetic models. (G) Correlation of model predictions and experimentally measured metabolite concentrations in the top 10 kinase mutants from (F). (H) The systems-nature of metabolism control: differential expression of a few individual pathway enzymes is sufficient to induce a redistribution of flux control among a broad set of enzymes. Fractions of differentially changed enzymes from the model are plotted on the x axis. The y axis shows the median change of control coefficient for each parameter comparing to the parental strain divided into 4 groups. Group (0.75, 1) has coefficients with the median change up to >100% in comparison to WT.

MCA was then used to assess how the observed rearrangements in enzyme levels interact with central metabolism. We generated a specific glycolytic model for each kinase knockout by adjusting enzyme concentrations in a highly curated glycolytic model (Smallbone et al., 2013) according to our measurements. Flux (*C*^*J*^*E*) and concentration control (*C*^*S*^*E*) coefficients were determined to measure the relative steady-state change in the global system variables, i.e., flux (J) or metabolite concentration (S), in response to differential enzyme expression (E) (Kacser and Burns, 1973). Differential enzyme expression altered the overall flux control coefficients (FCCs) by more than 50%, for two-thirds of glycolytic enzymes in 78% of the kinase knockout strains. Similarly, the enzyme abundance changes as measured, altered the overall concentration control coefficients by more than 50% (Figure 3A). Differential enzyme expression does hence redistribute the control over glycolytic flux between different metabolic enzymes. We illustrate this situation for the metabolic flux going through alcohol dehydrogenase (ADH_ADH1, reaction abbreviation were kept as in Smallbone et al. [2013]). In the WT situation, the highest control over ethanol production is attributable to glucose phosphorylation by *hexokinase 2* (*HXK2*) (Figure 3B). Due to differential enzyme levels, the flux control shifts to other enzymes in the mutants (Figures 3B and S11), altering steady state by more than 2-fold in 48% of the kinase knockouts. The model predicts that in 55% of the kinase mutants, this re-shuffling affects metabolite concentrations (Figure 3A insets).

A principal-component analysis (PCA) of the FCCs yielded four distinct clusters. The cluster division was mainly attributable to the control of *HXK2*, phosphofructokinase 2 *(PFK2)*, and *ADH1* on glycolysis and energy metabolism (Figure S12), of which glucose phosphorylation by *HXK2* was the most dominating (*HXK_GLK*1 flux) (Figures 3C, inset, and S12). This result is consistent with experimental observations. *HXK2* is a known regulator of *GLK1*, alternatively expressed under different carbon sources (Rodríguez et al., 2001). Indeed, the ratio of *HXK2/GLK1* expression differs between the clusters (Figure 3D). In cluster 2, *HXK* was more than two times lower expressed as *GLK1*. Despite being the strongest contributor, however, the ratio of *HXK2/GLK1* expression alone is not sufficient to explain the differences, underlining that even in central metabolism, differential enzyme expression acting in concert is required to explain metabolic regulation (Figure 3E). Therefore, we simulated the impact of multifactorial enzyme expression changes on glycolytic flux. Altering the expression level of as little as 7 enzymes, as detected in the kinase knockouts, can change the median of all control coefficients by up to 100% (Figure 3H). Taken together, these analyses predict that differential enzyme expression affects central metabolism significantly and mainly by redistributing flux control between different sets of enzymes.

To test the predictions, we used liquid chromatography-selective reaction monitoring (LC-SRM) to quantify ATP, ADP, and AMP; glycolytic and pentose phosphate pathway (PPP) intermediates; as well as amino acids and Krebs cycle metabolites (Figure S13; STAR Methods). On this set of central metabolites, 34 of the 97 kinase knockouts exhibited one or more strong concentration changes (± 2 SDs from mean concentration levels; Figure S14). These measured concentrations correlated significantly with the predictions (Figures 3F and 3G).

### Predicting the Metabolome from the Enzyme Expression Data

**Figure 4:**
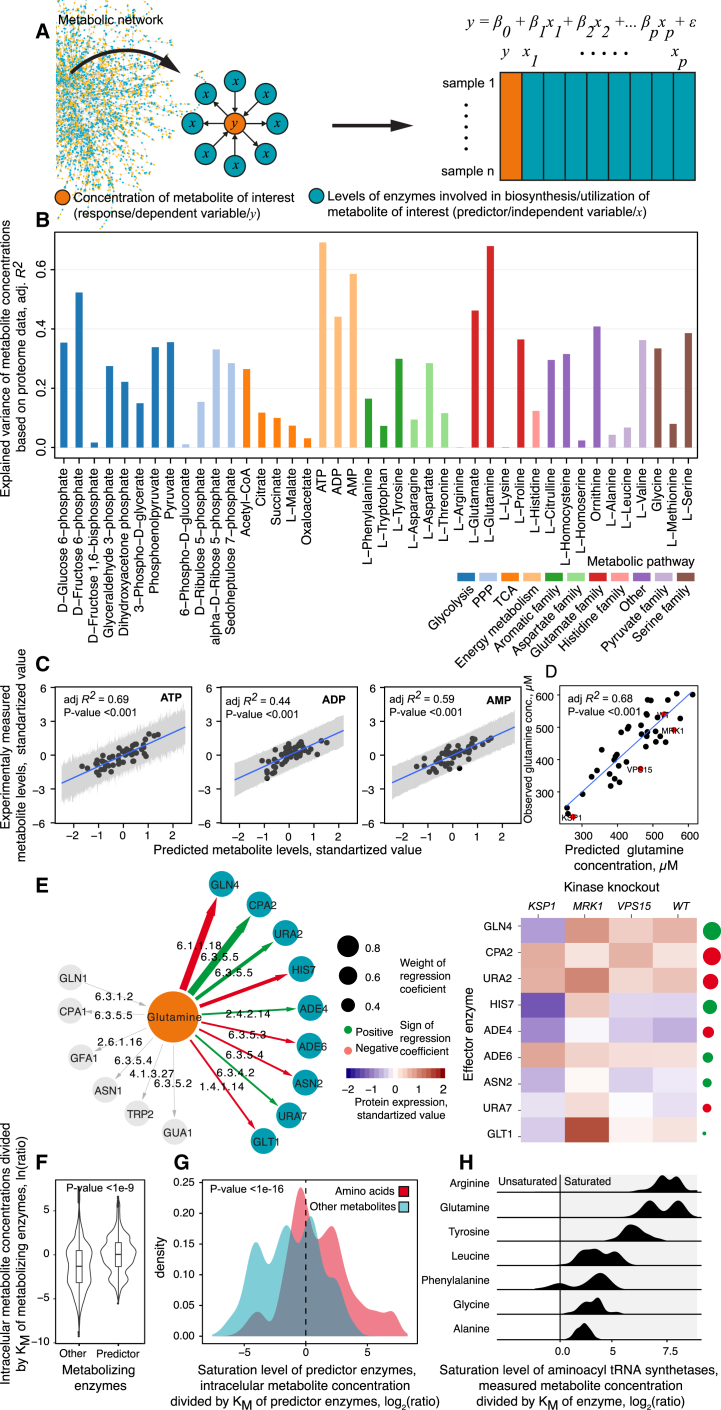
Multiple Linear Regression Identifies Multivariate Metabolite-Enzyme Relationships That Are Informative about Metabolite Concentration (A) Scheme: multiple linear regression (MLR) applied over the metabolic network topology to connect enzyme levels with metabolite concentrations. Metabolite concentrations (*y*) are expressed as a function of expression levels (*x*) of the closest enzyme neighbors in the metabolic network. Informative multivariate relationships between enzyme and metabolite concentrations are identified by exhaustive feature selection by computing all possible linear models and ranking them according to minimal Akaike information criterion (STAR Methods). (B) MLR reveals multivariate enzyme-metabolite relationships that explain metabolite concentrations in kinase knockouts. The bar plots indicate the coefficient of determination (adjusted R^2^) between predicted and experimentally determined metabolite concentrations across the kinase deletion strains. See also Figure S15. (C) The correlation of predicted and measured ATP, ADP, and AMP levels across kinase knockouts. x axis: predicted concentration from enzyme expression profiles, y axis: concentration as measured by liquid chromatography-selective reaction monitoring (LC-SRM). (D) The predicted and experimentally measured glutamine concentrations in kinase deletions correlate with an adjusted R^2^ = 0.68. Red dots highlight examples of enzyme expression patterns from (E) for representative in quartile of glutamine concentrations. (E) Left: graphical illustration of the 9 (out of 15) glutamine-metabolizing enzymes that are associated by the MLR approach to glutamine concentration. Right: as glutamine participates in multiple metabolic reactions, a correlation of the expression level of one glutamine-metabolizing enzyme at a time, as applied in many multi-omic studies, would fail to detect any correlation between enzyme expression and metabolism. (F) Enzymes that influence metabolite concentrations across kinase knockouts are more likely saturated compared to other enzymes connected to the same metabolites; *K*_*M*_ values, as obtained from BRENDA (Chang et al., 2015), are compared to the concentration of the metabolites as measured in our study by LC-SRM. The level of saturation is expressed as a ratio between metabolite concentration and the enzyme’s *K*_*M*_ value. (G) Enzymes that affect amino acid concentrations are more saturated compared to other enzymes associated with the rest of the metabolites. (H) Aminoacyl-tRNA synthetases, which are predictive of multiple amino acid concentrations, are typically saturated based on their *in vitro* kinetics.

Next, we asked whether proteomic data could be used to explain the variation metabolite concentrations also at the scale of the metabolic network. First, we generated a network graph connecting the metabolites to enzymes according to a genome-scale reconstruction of the yeast metabolic network (Herrgård et al., 2008). The 46 metabolites quantified connect as a substrate, product, or cofactor to 192 enzymes (= 1st order neighbors). Each of these metabolite-enzyme relationships was expressed as a multiple linear regression (MLR) problem (Figure 4A). Then, we used exhaustive feature selection and ranked all possible models (>10^12^) according to minimal Akaike information criterion (AIC) (Akaike, 1974). To minimize the risk of overfitting, we repeated the procedure 1,000 times for each metabolite using random subsets of the data and retained the top 5 most frequently identified metabolite-enzymes relationships from all random subsets (Figure S15), ranked them according to the best agreement between predicted and measured metabolite concentration, and calculated their explanatory power (as adjusted R^2^). The models with the best fit were diagnosed for outliers, influential observations, residual structure, and the presence of autocorrelation. All models that violated this set of criteria were discarded. The statistical models obtained in this way show that changes in enzyme abundance do explain metabolite concentration (Figure 4B). Fructose-6-phosphate, glutamate, glutamine, ATP, ADP, and AMP levels as estimated from enzyme abundance correlate with their experimentally measured concentrations (adj. R^2^ > 0.4; Figure 4B). We illustrate the model performance for ATP, ADP, AMP (Figure 4C), and glutamine, the metabolites for which the simple linear regression model provided the best results (Figures 4D and 4E). Thus, when accounting for metabolic network topology and multifactorial relationships, enzyme expression is informative of metabolite concentrations.

By being applied over the actual metabolic network topology, the feature selection approach identifies the predictive enzymes. For example, glutamine is connected to 28 of the quantified enzymes. Multiple feature selection identified 9 of them (*GLN4, CPA2, URA2, HIS7, ADE4, ADE6, ASN2, URA7*, and *GLT1*) to be significant contributors to its concentration (Figure 4E, in order of weight in the model), and together, their differential expression explains 68% (adj. R^2^) of the experimentally detected glutamine concentration changes (Figure 4D). The strongest predictor of glutamine levels was *GLN4*, the glutamine aminoacyl-tRNA synthetase, indeed already known to be important for glutamine regulation (Murray et al., 1998). Of note, MLR identified aminoacyl-tRNA synthetases as the strongest predictors also for aspartate, glycine, proline, and tyrosine (Figure S15). MLR hence confirmed that tRNA loading is a major factor in amino acid concentration regulation (Wegrzyn and Wegrzyn, 2008, Whitney et al., 2007) and revealed that it is quantitatively one of the strongest single contributors to amino acid concentrations in general.

Other illustrative examples are ATP, ADP, and AMP (Figure 4C) that are among the most connected metabolites (Zomorrodi and Maranas, 2010). From the 88 ATP, ADP, or AMP metabolizing enzymes quantified, 33 were found predictive about their levels (Figure S15). This list contains many of the high-flux enzymes associated with the cellular energy charge, including *HXK2*, subunits of the electron transport chain ATPase (Complex V, *ATP2*) or the vacuolar ATPase (*VMA1*), a major consumer of cellular ATP (Beyenbach and Wieczorek, 2006).

Predictive and non-predictive enzymes were not different in their average abundance (Figure S16) but were so in saturation (substrate concentration > K_M_) according to *in vitro* determined K_M_ values obtained from the Braunschweig Enzyme Database (BRENDA) (Chang et al., 2015). Considering the cell-average metabolite concentrations as measured in our study, enzymes identified by MLR were more than three times closer to the saturation than all other enzymes connected to the same substrates (Figure 3F; Wilcoxon rank-sum test, p < 10^−16^). 45% of these enzymes appear saturated. Moreover, 40% of metabolites were associated with at least one enzyme with a K_M_ value at least ten times below the metabolite concentration. Amino acid metabolizing enzymes were >8-fold (comparing medians) (Wilcoxon rank-sum test, p < 10^−16^) more saturated than predictors for other metabolites (Figure 4G). In accordance with the MLR analysis (Figure S15), we find that aminoacyl-tRNA synthetases were among the most saturated enzymes (Figure 4H).

**Figure 5:**
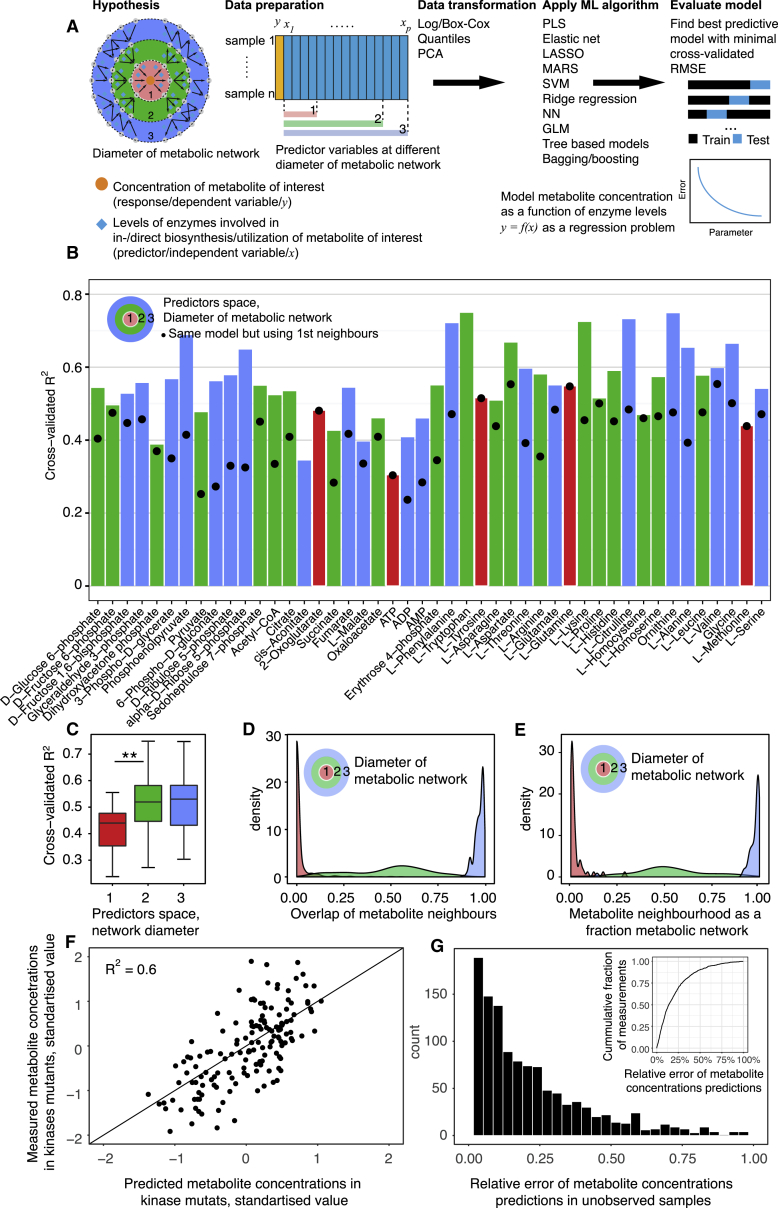
Machine Learning Regression Predicts the Concentration of Metabolite Pools from Enzyme Abundance (A) Scheme: mapping the dependency of metabolite concentrations on enzyme expression levels by incorporating the structure of the metabolic network in a genome-scale application of machine learning (ML). Different data transformation techniques and twelve ML algorithms were applied over the metabolic network topology, and the obtained models were ranked according to their ability to predict metabolite concentrations from the enzym abundance (expressed as minimal cross-validated root-mean-square error [RMSE]). In comparison to MLR (Figure 4), the inclusion of ML enabled network expansion to the 2^nd^ and 3^rd^ order neighbors, upon which enzyme expression changes across the full metabolic network are incorporated (E). (B) ML enables the predictions of metabolite concentrations in the kinase knockouts on the basis of the enzyme abundances measured. Shown is the correlation of measured metabolite concentrations in relation to the predicted metabolite concentrations, expressed as 10-fold cross-validated R^2^. The median cross-validated R^2^ is 0.549, implying that at least half of metabolite concentration changes are explained by changes in enzyme abundance. The dots indicate the predictive power achieved with the directly metabolizing enzymes; the color indicates whether maximal predictability was reached upon including 1^st^, 2^nd^ or 3^rd^ order enzyme neighbors. (C) For most metabolites, the predictive power is concentrated within the directly metabolizing enzymes (1^st^ order neighbors) or is partially improved upon incorporating also the 2^nd^ order neighbors. Ruling out overfitting, the predictions did not improve upon further expansion of the predictor variable space to the full metabolic network. ^∗∗^ = Wilcoxon rank sum test p value < 0.01. (D) The commonality of enzyme predictors for the different metabolites, accounting for network diameter, reveals a spectrum of enzyme expression signatures that can regulate metabolite abundance. (E) The total fraction of enzymes associated with metabolite concentrations accounting for network distance. (F) Metabolic phenotype (all metabolites per mutant) predictions by ML in unobserved kinase knockout strains on the basis of their quantitative proteome. The phenotype prediction is based on individual metabolite models; the top 30 predicted kinase metabolomes are shown. (G) Distribution of relative errors (in %) in the prediction compared to experimental measurements of metabolite concentrations in all kinases knockout strains; ML predicts metabolite concentrations accurately.

In order to make use of the full metabolic enzyme expression matrix to predict metabolite concentrations, we implemented a pipeline that makes use of 12 ML algorithms. This analysis is summarized graphically in Figure 5A and detailed in the STAR Methods. In brief, we reduce the dimensionality of the proteome dataset, divide the data into training and testing sets using cross-validation to obtain best predictive regression model for each metabolite. Our ML approach predicted metabolite concentrations that on average correlated with the measured concentration values with a cross-validated R^2^ value of 0.55. The highest predictability from enzyme abundance was revealed for tryptophan, ornithine, and citrulline, for which the predictions correlated with cross-validated R^2^ of 0.75, 0.75, and 0.73, respectively, with their experimentally measured concentrations (Figure 5B).

The size of the metabolite’s network neighborhood had only minimal influence on predictability. Several of the metabolites (ATP, 2-oxoglutarate, tryptophan, glutamine, and methionine) remained exclusively predicted by their directly metabolizing enzymes, (Figure 5C). For all other metabolites, predictability increased upon incorporation of the second-order neighbors but not anymore upon further network expansion (Figure 5C). Metabolite concentrations are therefore most sensitive to enzyme abundance changes occurring in their immediate neighborhood (Figures 5D and 5E). Of note, the best overall performing ML algorithm on our dataset was ridge regression with greedy variable selection (Zhang, 2011) (Figures 5B and S17). Finally, we tested the power of the ML model to predict not only the individual metabolites but also entire metabolomes. For this, we repeated the whole procedure ninety-seven times, using leave-one-out cross-validation for the entire dataset. The metabolomes predicted agreed with the metabolomes as measured experimentally (Figure 5F): 70% of absolute metabolite concentrations were predicted with less than 25% relative error (Figure 5G).

**Figure 6:**
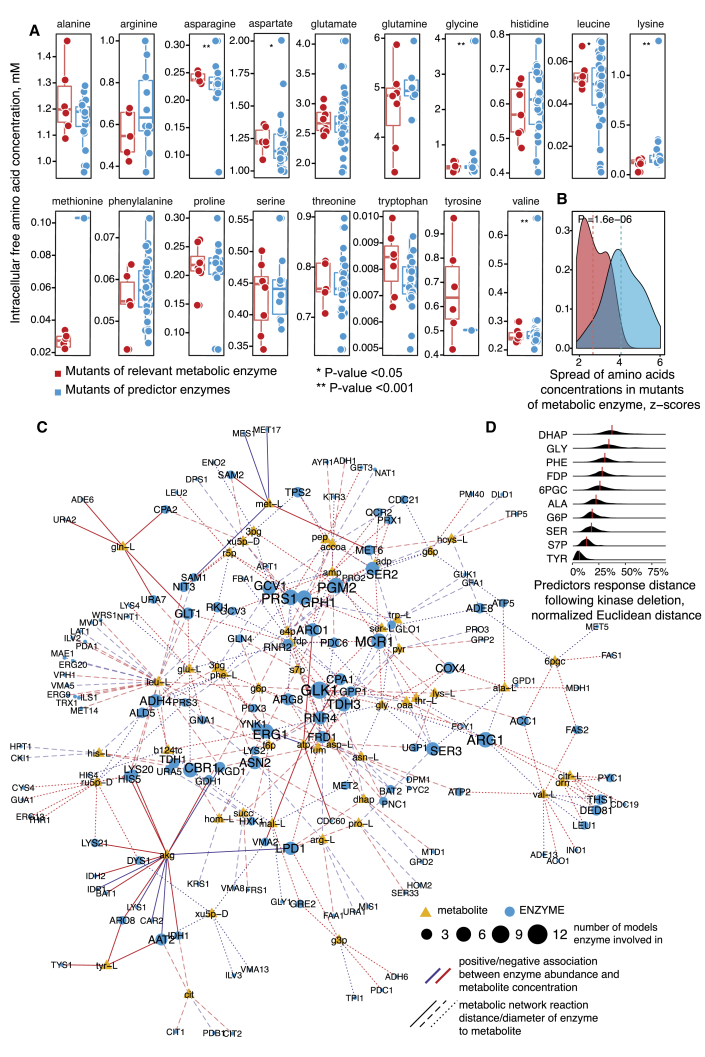
Machine Learning Trained over the Metabolic Network Topology Reveals Genes and Metabolites Important for Metabolite Concentration Regulation (A) Enzymes whose abundance predicts metabolite concentrations in kinase knockouts cause metabolite concentration changes when deleted in a completely independent dataset (Mülleder et al., 2016a). (B) Summary of (A): the overall range of metabolite concentration changes is broader upon the deletion of enzymes associated with concentration changes, as it is upon the deletion of all other enzymes that convert the same metabolites. (C) Enzyme metabolite graph depicting hub proteins in the prediction of the yeast cell metabolome. Nodes represent metabolites (triangles) that are predictable using relevant enzyme abundances (circles). Edges represent positive and negative association represented by Pearson’s correlation between metabolite and enzymes levels. For visualization purposes, we retained only the most important enzymes (normalized weight of variable >90%, with up to 5 enzymes with highest absolute loading per component). (D) The concentration of several hub metabolites is affected by a spectrum of enzyme expression signatures, while for some metabolites only specific expression signatures were observed. More distant values (upper density plots) illustrate situations where a (kinase-deletion) unique combination of enzyme expression changes affects a particular metabolite. Contrarily, lower distances illustrate cases where multiple kinase deletions affect a metabolite via the same set of enzyme expression changes. The GAPDH substrate DHAP was the metabolite controlled by the highest number of divergent mechanisms, while tyrosine was the most uniformly regulated metabolite (for illustration purposes, only every 5^th^ metabolite is depicted; the full figure is provided in Figure S18). To compare predictor responses between metabolites, the levels of associated enzymes were standardized (to zero mean and unit variance). The Euclidean distance of standardized enzyme expression was computed pairwise between each kinase mutant and normalized to 100% by the most distant kinase pair. Red vertical lines denote the median value for each enzyme. Abbreviations: amino acids are given in three letter IUPAC code; DHAP, dihydroxyacetone phosphate; FDP, Fructose 1,6 bisphosphate; 6PGC, 6-phosphogluconate; G6P, glucose 6-phosphate; S7P, sedoheptulose 7-P.

To test the validity of the predictions also on an independently generated dataset, we made use of amino acid concentrations that have been determined upon the systematic deletion of all non-essential yeast genes (Mülleder et al., 2016a). We compared the range of amino acid concentration changes, measured upon the deletion of (1) the subset of non-essential enzymes for which ML had attributed an important regulatory role (defined as >50% maximum weight of predictor variable; see STAR Methods) and (2) the rest of the enzymes that metabolize the same amino acids. ML had correctly identified genes whose deletion affected affect amino acid concentrations (Figure 6A, Bartlett’s test, symbols ^∗^ and ^∗∗^ correspondingly denote p < 0.05 and p < 0.01, respectively; Figure 6B, Wilcoxon rank-sum test, p = 1.6e−06). Hence, on the basis of enzyme abundance, ML is able to estimate entire metabolomes as well as identify genes important for the cell’s metabolic phenotype.

### Interpreting the Machine Learning Models to Draw Genotype-Phenotype Maps

We made use of the metabolic network topology to interpret the ML models and to gain insights into the biological mechanisms. Each metabolite was connected to the top 5 loading enzymes of highly predictive principal component features (90% of highest predictors weight) to reveal enzymes with the most active role (Figure 6C). The obtained graph reveals that some of the most active regulators, including *glucokinase (GLK1), phosphoglycerate mutase (PGM2), glyceraldehyde 3-phosphate dehydrogenase* (*GAPDH* gene *TDH3*), and *argininosuccinate synthetase (ARG1)*, exert distal regulation over multiple metabolite concentrations. In particular, glucokinase *GLK1* levels are associated with concentration changes in many metabolites, including aspartate, leucine, and glycine. *GLK1* was positively associated (*r* = 0.38, p = 0.00511) with the tricarboxylic acid (TCA) cycle metabolite oxaloacetate, indicating the coordinated regulation of glycolysis and the TCA cycle. In contrast, *GLK1* expression was negatively associated with aspartate, glycine, and threonine concentrations (*r* = −0.49; −0.46; −0.38, p = 0.000762; 0.00158; 0.0115, respectively). Hierarchical metabolite regulation by *GLK1* and *PGM2* expression has also been independently identified using Bayesian analysis (Bradley et al., 2009). Another example is the identification of *GAPDH*, in which abundance or activity changes have been shown to regulate the PPP to achieve yeast redox balance (Grüning et al., 2011). Indeed, a PCA of all metabolic changes detected reveals PPP metabolites to be the strongest separator; changes in the PPP are the most frequent metabolic response in kinase knockouts (Figure S14).

Finally, we assessed to what extent enzyme expression patterns are recurrent to explain changes in the metabolome. Comparing the profiles of predictive enzyme expression changes, we obtain a wide range of regulation patterns, ranging from specific to general patterns. For example, the enzyme expression landscape leading to a concentration change in dihydroxyacetone phosphate, phosphoenolpyruvate, leucine, and acetyl-CoA were substantially specific to each of the kinase knockouts (Figures 6D and S18). On the other hand, the enzyme expression landscape associated with concentration changes in amino acids such as tyrosine, methionine, and ornithine was observed in multiple knockouts (Figures 6D and S18)

## Discussion

Here, we address an apparent contradiction in the current literature about the regulation of metabolism. On the one hand, many investigations attribute an important role to gene expression in the regulation of metabolism. On the other hand, the prediction of metabolomes from gene expression data has so far proven challenging, and several multi-omic studies reported low correlation values of enzyme expression and metabolite levels as well as fluxes (Chubukov et al., 2013, Daran-Lapujade et al., 2007, Fendt et al., 2010, Millard et al., 2017). So how can gene expression regulation be of utmost importance for metabolism and at the same not be correlated with metabolism and not explain metabolite levels?

The reason for this discrepancy is implied by a Gedankenexperiment, in which metabolite levels are calculated upon changing enzyme abundance values in a kinetic model of glycolysis (Smallbone et al., 2013). In this hypothetical simulation, all metabolite concentration changes are caused by enzyme abundance changes. Yet, a typical correlation analysis would have yielded low scores (cophenetic correlation coefficient = 0.35) between enzyme abundance and metabolite concentration (Figure S19). In contrast, the metabolite concentrations were highly correlated with the calculated fluxes (cophenetic correlation coefficient > 0.8, Figure S20). In other words, even in this theoretical simulation in which metabolite concentration changes are fully caused by enzyme abundance changes, consistent with previous reports (Hackett et al., 2016, Millard et al., 2017), the one-to-one mapping would report low correlation values between enzyme abundances and metabolite concentrations (Figures S21 and S22). Taken the other way, a low one-to-one correlation between enzyme and metabolite abundance is not due to metabolite concentrations that would behave independently to changes in enzyme expression; the low values are the consequence of the more complex, multifactorial relationships that describe the interdependence of enzyme abundance and metabolite levels.

Our results show that metabolic gene expression regulation is achieved through many enzyme expression changes acting in concert. Once these multifactorial relationships are identified—in our study through the use of multivariate statistical learning—enzyme expression landscapes become predictive about the cellular metabolome, even at the network scale, that is currently not to be covered by mechanistic models as they are available, i.e., for individual metabolic pathways, such as glycolysis. To our knowledge, this is the first successful attempt to predict a complex, quantitative metabolic phenotype from enzyme expression without taking into account predetermined enzyme reaction mechanisms, phosphorylation states, or kinetics. Applied over the topological organization of the metabolic network, the predictive models are further rendered interpretable, which, as we have shown, enables to draw genotype-phenotype maps. Taken together, these results demonstrate that enzyme expression landscapes are regulated to control metabolite concentrations and as a consequence, fluxes. On average, the metabolome predictions achieved on the basis of enzyme levels correlated with experimental values with a cross-validated R^2^ of 0.55. This suggests that more than half of metabolite concentration regulation, at least as observed in kinase knockouts, is attributable to changes in enzyme abundance.

## STAR★Methods

### Key Resources Table

**Table:** 

REAGENT or RESOURCE	SOURCE	IDENTIFIER
**Chemicals, Peptides, and Recombinant Proteins**		

Acetonitrile UPLC grade	Greyhound BIOSOLVE	Cat# Bio-012041
Ultra-pure water, ULC-MS grade	Greyhound BIOSOLVE	Cat# 23214125
Methanol Absolute ULC-MS grade	Greyhound BIOSOLVE	Cat# BIO-13684102
Ammonium formate	Fluka	Cat# 14266
Formic acid	Fluka	Cat# O6454
L-Amino acids analytical standard	Sigma-Aldrich	Cat# LAA21
Standards for glycolysis, TCA and PPP intermediates	Sigma-Aldrich	Cat# G8270, G7879, F3627, F6803, G5251, P8877, 79470, 79470, P7127, P2256, P7877, 83899, R7750, 15732, 78832, W302600, A3412, 75892, S3674, 47910, 27606, O4126
Standards for cofactors	Sigma-Aldrich	Cat# N8229, N7004, N5130, 93220, A2754, A2383, 01930, A2056

**Critical Commercial Assays**		

Retention time peptides Biognosys iRT kit	https://biognosys.com/	Ki-3002-1

**Deposited Data**		

Raw proteome data	This study	PRIDE: PXD010529
Processed proteome and metabolome data	This study	[https://doi.org/10.5281/zenodo.1320288]
*S. cerevisiae* genome-scale metabolic reconstruction	Herrgård et al. (2008)	N/A
*S. cerevisiae* kinetic glycolysis model	Smallbone et al. (2013)	BioModels: MODEL1303260018
Km values from BRENDA database	Chang et al., 2015, Schomburg et al., 2000	https://www.brenda-enzymes.org/
Curated genetic information, gene ontology, literature and phenotype annotation from the Saccharomyces Genome Database (SGD)	Cherry et al. (2012)	http://www.yeastgenome.org/
Yeast kinase signaling pathway annotations from Reactome	Fabregat et al. (2016)	https://reactome.org
Yeast kinase signaling pathway annotations from KEGG	Kanehisa et al. (2016)	https://www.kegg.jp/kegg/rest/keggapi.html
Amino acid concentrations in metabolic enzyme knockouts	Müelleder et al. (2016a)	https://doi.org/10.17632/bnzdhd6ck8.1
Yeast protein-protein interaction network	Szklarczyk et al. (2015)	https://string-db.org/
Yeast protein degradation rates	Christiano et al. (2014)	https://ars.els-cdn.com/content/image/1-s2.0-S2211124714009346-mmc3.xlsx

**Experimental Models: Organisms/Strains**		

Prototrophic *Saccharomyces cerevisiae* kinase deletion collection (*MAT*a, prototrophy restored episomally)	Winzeler et al., 1999, Mülleder et al., 2012	http://www.euroscarf.de/
Selected *Saccharomyces cerevisiae* kinase deletion strains (*MATα*, prototrophy restored episomally)	Winzeler et al. (1999)	http://www.euroscarf.de/
**Recombinant DNA**		

Plasmid: pHLUM	Müelleder et al. (2016a)	In addgene.org: #40276
Plasmid: pHLU	Müelleder et al. (2016a)	In addgene.org: #64181
Plasmid: pHLUK	Müelleder et al. (2016a)	In addgene.org: #64167

**Software and Algorithms**		

Scripts to reproduce main figures	This study	https://github.com/zelezniak-lab/kinase_metabolism
Proteomics data analysis Spectronaut (8.0.9600)	Biognosys	Sw-3001
Proteomics data analysis via Deep Neural Networks, DIA-NN	Demichev et al. (2018)	https://github.com/vdemichev/DiaNN
caret R package (6.0-78) for regression modeling	Kuhn (2008)	http://topepo.github.io/caret/index.html
LP solver IBM ILOG CPLEX Optimization Studio 12.7.1 for flux coupling analysis	IBM	CJ1HQML
libRoadRunner (1.4.8) for metabolic control analysis	Somogyi et al. (2015)	https://github.com/sys-bio/roadrunner
MassHunter software suite for metabolite analysis	Agilent Technologies	N/A
grofit R package (1.0) for growth curves analysis	Kahm et al. (2010)	http://CRAN.R-project.org/package=grofit
sva R package (3.26.0) for batch correction of data	Leek et al. (2012)	https://doi.org/10.18129/B9.bioc.sva

### Contact for Reagent and Resource Sharing

Further information and requests for resources and reagents should be directed to and will be fulfilled by the Lead Contact, Markus Ralser (Markus.Ralser@crick.ac.uk)

### Experimental Model and Subject Details

#### Strains and Culture

Yeast strains used in this study were obtained from our published prototrophic gene deletion collection (Mülleder et al., 2012). Kinases were identified following the strategy of Sharifpoor et al. (2012), expanded by the annotation in the yeast kinome and yeast genome database (Breitkreutz et al., 2010, Cherry et al., 2012) and including genes associated to Gene Ontology term 0004672 (protein kinase activity). 97 of the strains grew in triplicates (n=3) in minimal medium without a substantial growth defect (Figure S1), were pre-cultured overnight in 10 ml minimal medium, at 30°C, and diluted them to an OD_600_ of 0.2 in 30 ml main culture.

Growth was monitored, and the strains were sampled by cold methanol quenching at an OD_600_ 1.5 +/- 0.1, before the cultures enter the diauxic shift, for metabolic and proteomic analysis. The growth curves were fitted using non-parametric (without growth law assumption) spline model as implemented in R growFit package (Kahm et al., 2010). Exponential growth rate was estimated as maximal slope of the growth curve (Figure S1).

To generate heterozygous and homozygous diploid strains of the kinase mutants we inoculated the respective *MATa* and *MAT*α strains in 150 μl YPGlucose (2%) medium and incubated them overnight before 2 consecutive selection steps on synthetic complete medium (SC) lacking lysine and methionine. For heterozygous strains a *HIS3* deletion strain of the opposite mating type was selected. Prototrophy was restored in the haploid parents and the diploid progeny by transformation with a single copy plasmids containing the required genes (pHLUK, pHLUMv2 and pHLU (Mülleder et al., 2016b). All 5 versions of 10 randomly chosen kinase mutants (the two parental haploid MatA and Matα, 2 heterozygous and 1 homozygous diploid) strains were grown on synthetic minimal (SM) and collected in exponential phase.

### Method Details

#### Metabolomics

Free intracellular metabolite pools were quantified by liquid chromatography - selective reaction monitoring (LC-SRM) by protocols described previously. The method used to obtain Dataset 1 (Figure S13) is described in Keller et al. (2014) for the quantification of glycolytic and pentose phosphate pathway metabolites and was expanded with additional transitions for ATP, ADP and AMP. Analytes were separated by gradient elution using 10% and 50% acetonitrile, containing 750 mg l^−^^1^ octylammonium acetate as solvents A and B at a flow rate of 0.6ml/min and column temperature of 20°C. The gradient program was as follows: 5% B for 3.5 min, then ramping to 70% B within 2.5 min, followed by washing with 80% B for 0.5 min and re-equilibration at 5% B for 0.5 min, resulting in a total cycle time of 7.5 min on a Zorbax SB-C8 Rapid Resolution HD, 2.1x100mm, 1.8 Micron (Agilent) column.

For Dataset 2 and 3 we adapted chromatographic parameters from Buescher et al. (2010) and added a SRM set that has previously been established by individually optimising ion optics and fragmentation settings using commercially available standards on an 6460 or 6470 Triple Quadrupole Mass Spectrometer (Agilent) coupled to UPLC (1290 Infinity, Agilent).

In Dataset 2 analytes were separated by gradient elution using 10 mM TBA 15 mM acetic acid in water and 5% methanol as solvents A and B at a flow rate of 0.5 ml/min and column temperature of 30°C. The gradient program was as follows: 0% B for 4.5 min, increase to 20% B (5 min), 70% B (9.5 min), 90% B (10 min), kept constant until 12 min and returned to initial conditions at 12.5 min followed by 1.5 min equilibration, resulting in a total cycle time of 14 min on a Zorbax Eclipse Plus C18 2.1x50 mm, 1.8 μm column (Agilent).

In Dataset 3 (Figure S13), free amino acids were separated by hydrophilic interaction liquid chromatography (HILIC) using an ACQUITY UPLC BEH amide column (130Å, 1.7 μm, 2.1 mm X 100 mm) by gradient elution at a constant flow rate of 0.9 ml/min and a column temperature of 25°C. Eluents A and B were prepared at 10 mM ammonium formate, 0.176% formic acid and in 95/5/5 acetonitrile/MeOH/water and in 50/50 acetonitrile/water respectively, all of UPLC grade. Chromatographic conditions for the gradient elution were following: solvent A was kept for 0.7 min at 85% before a steady decrease to 5% A until 2.55 min. A was kept at 5% for 0.05 min before returning to the initial conditions of 85% A within 0.05 min. This was followed by and an equilibration step until 3.25 min before injection of the next sample. All metabolites were identified by matching retention time and fragmentation pattern with the commercially obtained standards and were quantified by external calibration (except Dataset 1) with standards prepared at serial dilution from 500 μM to 100 nM.

Dataset 1 was created from the same cells as grown for the proteomic experiments. Metabolomics datasets 2 and 3 were obtained by re-growing 3 independent cultures from strains with highly variable metabolite concentrations based on dataset 1 and a previous genome-scale metabolism study (Mülleder et al., 2016a). Mass spectrometry signals for all metabolites were acquired in dynamic SRM mode in Masshunter software. All preprocessed metabolomics data (integrated SRM transition peaks after external calibration (where applicable)) were corrected for batch effects using *ComBat* approach as implemented in *sva* (Leek et al., 2012) R package. For visualization purposes (Figure S14) missing metabolite concentrations were imputed using *amelia* approach (Honaker et al., 2011).

#### Proteomics

The proteomic method has been published in parallel (Vowinckel et al., 2018). In brief, tryptic digests for the analysis by SWATH-MS were prepared by the RapiGest method as described previously (Vowinckel et al., 2013), and analysed on on a Tandem Quadrupole Time-of-Flight mass spectrometer (SCIEX TripleTOF5600) coupled to DuoSpray Ion Source (SCIEX) and Eksigent 425 HPLC system running in microflow mode. Before the injection into the mass spectrometer, total protein concentrations were adjusted by dilution. SWATH assay libraries were built following Schubert et al. (2015) by pre-fractionation of the tryptic digest. Unless otherwise indicated, SWATH data quantification was performed in Spectronaut (Biognosys, v. 8.0.9600). Post-processing was conducted in R (R Core Team, 2015) by first removing precursors from all samples where the median peak group Q-value was > 0.01 obtained from mProphet algorithm as implemented in Spectronaut. For label free quantification, we considered only the precursors originating from uniquely mapping peptides. Next, we chose peptides by correlation quantity following our approach as developed previously (Alam et al., 2016, Vowinckel et al., 2018). This strategy assumes that the best quantitation-informative peptides, as they are derived from the same protein, correlate in their abundance. Pearson’s correlation coefficients were calculated between each pair of peptides (summed precursor's MS2 peak areas) belonging to the same protein across all samples. Peptides displaying overall low correlation (<0.3) were removed from subsequent analysis. This selection therefore excludes non-specific peptides or precursors which are not linearly responsive for other reasons, e.g. due to post-translational modifications. Furthermore, to account for confounding effects related to acquisition dates, we performed batch correction using the *sva* approach (Leek et al., 2012). Supervised surrogate variable analysis (with 1 variable) was applied without specifying experimental factors (Nygaard et al., 2016) using 50% of least varying peptides as controls. Estimated surrogate effects were regressed out from the peptide signal. Finally, for each protein, the signals of all peptide groups were geometrically averaged. External standard quality control (QC) samples were prepared as a mixture of all proteomes and were measured every 8-12 injections. After applying batch correction, QC samples are clustered together around 0 on a scaled PCA plot, showing that the batch correction strategy has removed most of the confounding effects Figure S2.

Diploid strains were analysed with a slightly modified proteomics workflow (Demichev et al., 2018). Briefly, proteins were extracted in 6M urea/ 0.1M ammonium bicarbonate using a bead beater (Spex Geno/Grinder). After reduction and alkylation with dithiothreitol (5mM) and iodoacetamide (10mM), respectively, proteins were digested overnight with trypsin. The resulting peptides were cleaned-up using 96-well MACROSpin plates (Nest Group). Samples were measured on a Waters nanoAcquity coupled to a SCIEX TripleTOF 6600. The peptides were separated with a 20min gradient on a Waters HSS T3 column (300um x 150mm, 1.8um) using a flow rate of 5ul/min. SWATH MS/MS acquisition scheme with 40 variable size windows and 35ms accumulation time was used. Raw data were processed with DIA-NN (version 1.2) using the default settings and mass accuracy set to 20 ppm and 12 ppm at the MS2 and MS1 level, respectively.

#### Enzyme Expression Analysis

After correction for batch effects, differential protein expression analysis was performed using *limma* (Smyth, 2005). Geometrically averaged fold-changes as outputted by *limma* were omitted, instead fold-change ratio of mean signals between mutant and parental strain were used throughout the manuscript. The Benjamini-Hochberg (BH) false discovery rate (FDR) control procedure (Benjamini and Hochberg, 1995) was applied after performing all comparisons using *p.adjust* as implemented in R-core *stats* package. Additionally, we used a cut-off of 40% change noted as log_2_(fold-change) of ±0.485 which we refer over the manuscript as log_2_(1.4/0.714) up-/down regulated empirically determined cut-off to account for any potentially unaccounted batch-to-batch variation left to further eliminate any potential false discoveries. For this, we calculated protein expression fold-changes for each protein in the QC sample (a mixture of all samples) and identified a tiny fraction of proteins that was significantly (adj P-value < 0.01) differentially expressed between different batches. The fold-change exceeding the median obtained from the distribution of these extreme cases was used as the cut-off throughout the analysis.

To estimate enzyme copy numbers, we compared two datasets recorded by fluorescence microscopy (Ghaemmaghami et al., 2003) and one by mass spectrometry (Kulak et al., 2014), showing overall agreement of enzyme copy number fraction of the total yeast proteome (∼35% and ∼36% respectively). To calibrate the relative abundance changes (Figure 1E) for each mutant, every enzyme’s copy number (Kulak et al., 2014) was multiplied by the fold-change if it was differentially expressed (BH adjusted p-value <0.01) compared to a WT strain. We then calculated the percentage change of total enzyme copy numbers in mutant comparing to the parental strain.

#### Flux Coupling Analysis

To identify active metabolic reactions, the reactions that are important for cellular growth, we performed flux coupling analysis (FCA) (Burgard et al., 2004) under several growth conditions, i.e. minimal media conditions, synthetic complete with and without oxygen. Physiological data for constraints were obtained from Van Hoek et al. (1998). Simulations were performed using an improved iMM904 model (Zomorrodi and Maranas, 2010). Reactions that were fully or partially coupled to biomass growth were considered as active. The flux coupling analysis procedure was implemented in C++ and solved using the IBM ILOG CPLEX Optimization Studio 12.7.1.

#### Metabolic Control Analysis

Metabolic control analysis was performed on the basis of the *S. cerevisiae* kinetic model (Smallbone et al., 2013), which was downloaded from the BioModels database (http://www.ebi.ac.uk/biomodels-main/) under ID MODEL1303260018. Enzyme abundances in the model were adjusted by multiplying original model's enzyme values by kinase mutants enzyme fold-changes, considering only significantly changed enzymes (BH adjusted p-value < 0.01), resulting in a model for every mutant. The steady-state simulations and calculations of control coefficients were performed with libRoadRunner (Somogyi et al., 2015) using the Python API. Metabolites and fluxes were considered to be in steady-state if there was less than a 10^-6^ increment in the solution. The overall flux control coefficients were calculated as described in Millard et al. (2017), i.e. taking for every enzyme the second norm over all its concentrations/flux control coefficients that were parameterised on it, e.g. for flux $CJ_{E}^{overall}=\sqrt{\sum_{j}{\left(C_{E}^{J}\right)}^{2}}$ analogously, overall concentrations control coefficients were calculated using $CC_{E}^{overall}=\sqrt{\sum_{s}{\left(C_{E}^{S}\right)}^{2}}$.

Metabolic regulation clusters were identified by first transforming all flux control coefficients across samples into PCA space, then calculating Euclidean distance using the first 10 components (retaining over 90% of variance) and performing Ward’s hierarchical agglomerative clustering. The number of cluster was then identified using two independent graphical methods “dindex” and “huber” as implemented in NbClust R package (Charrad et al., 2014).

#### Statistical Modelling

##### Data Preparation

Depending on the metabolomics experiment (Figure S13), we assigned a proteome measurement of matching genotype to each metabolite sample. For Dataset 1, replicates of proteomics experiments were averaged per genotype. For Datasets 2 and 3, where multiple biological replicates were available, we assigned random proteome measurements to the corresponding genotype. Then we only kept metabolic enzymes, as annotated in genome-scale yeast metabolic network reconstruction (Herrgård et al., 2008). Next, the metabolic network was converted to a bipartite metabolite-enzyme graph. Based on metabolic network topology, we selected enzyme neighbours at various metabolite network neighbourhood radii (Figures 4 and 5) for each measured metabolite. In total, for each modelled metabolite we created 3 response-predictor data matrices corresponding to different metabolic network radii. These were then used as basis for modelling of metabolite concentration data. For this, we used the batch corrected label-free protein quantifications and metabolite concentration measurements. Network manipulations were performed by calling routines from *igraph* library R package (Csardi and Nepusz, 2006).

##### Data Transformation

To each of the response-predictor matrices, a combination of data transformation methods were applied, specifically quantile normalization (Smyth, 2005), log-transformation, Box-Cox (Sakia, 1992) for predictors (enzyme levels), and log and Box-Cox transformations for responses (metabolite levels/concentrations). Both predictors and responses were standardized to have zero mean and unit variance. To reduce the dimensionality within the predictor space, predictors were transformed onto principal component space (PCA) for metabolite concentration modelling by machine learning. The choice of of number of principal components to retain was based on cumulative coverage of 99% of predictors variation. All data transformations were performed either using R base functions or the *preProcess* function as implemented in *caret* R package (Kuhn, 2008).

#### Metabolite Concentration Regression Modelling

The computational analysis is divided in the use of multiple linear regression (MLR), ‘explanatory part’, Figure 4), and using machine learning regression algorithms (‘predictive part’, Figures 4 and 5).

MLR modelling with exhaustive feature selection mainly was applied for exploratory purposes to identify readily-interpretable biologically meaningful associations while more advanced regression algorithms were used for metabolite concentration predictions. Model selection for MLR case was performed using the following procedures:

For each metabolite sample (without replacement) we created 1000 random subsets, each of them having 90% of the original data.

1) For each subset, we exhaustively evaluated all possible multiple regression models and chose the one with minimum Akaike information criterion (AIC) (as implemented by *regsubsets* function from the *leaps* R package) (Lumley and Miller, 2004)
2) We kept the top 5 most frequent models among all subsets
3) and remove outlier points based on Studentized residuals, exceeding Bonferroni adjusted p-value < 0.05 as implemented in *outlier Test* function in the car R package (Fox and Weisberg, 2011).
4) We removed influence points if any exceeded Cook’s distance thresholds, as calculated based on 4/(N−k−1), where N is the number of observations and k is the number of explanatory variables.
5) We tested for the presence autocorrelation using Breusch-Godfrey test (Bonferroni adjusted p-value < 0.05) (as implemented in *bgtest* function in *lmtest* package (Zeileis and Hothorn, 2002);
6) and determined how the obtained models explain the data by calculating the adjusted R^2^ value to assess the model fit.
7) To account for finite sample size, AIC was calculated according to the formula N^∗^log(RSS/N) + 2^∗^k (Faraway, 2016), where N is the number of observations, k is the number of explanatory variables, RSS is the residual sum of squares of linear model. Such ranking is not based on hypothesis testing, wherefore does not require FDR correction (Faraway, 2016). Models displaying the highest adjusted R^2^ are presented in the main text (Figure 4), the rest of the candidate models are present in Figure S15.

For MLR in the explanatory part, we used only the expression of the first enzyme neighbours of metabolites as features for the metabolite concentration modelling. Scaling was applied to predictors and responses without applying PCA transformation with exception of ATP where the number of predictors exceeded the number of samples. For machine learning (ML) regression (predictive part), we tested 12 algorithms. These are a generalised linear model with stepwise AIC feature selection, ridge regression with foba sparse learning algorithm, partial least squares regression, elastic net regression, lasso, multivariate adaptive regression spline, support vector machine regression, model averaged neural network for regression, recursive partitioning tree, bagged recursive partition tree, conditional inference tree and tree with stochastic gradient boosting. These ML methods were combined with the all possible data transformation strategies as described above. To identify the best predictive model, for each metabolite and data transformation, we optimised each model’s hyperparameters by retaining the model having the minimal average 100 times repeated 10-fold cross-validated root-mean-square error between the prediction and metabolite concentration measurement. The model’s hyperparameters were optimised using the unified *caret* interface (Kuhn, 2008). Then, the algorithm and the combination of data transformations demonstrating the best predictive performance was expressed as cross-validated R^2^ and used to compute metabolite concentrations with the all the proteomic data as input. Each metabolite was finally assigned the algorithm demonstrating the best predictive performance (Figure S17). Source code with grid ranges of hyperparameters for each algorithm are available through GitHub (https://github.com/alzel/regression_models).

The importance of variables was estimated by calling *varImp* function as implemented in *caret* R package (Kuhn, 2008). Variable importance is dependent on the particular algorithm (Kuhn, 2008). Coefficients are scaled to 100% based on the most important variable. In the present analysis, variables are principal components of the enzyme abundance matrix and we considered the variable to be important if it had an importance coefficient >50% and up to 10 enzymes with the highest absolute loading from each of the the components were chosen. In Figure 6C, for visualization purpose we displayed only enzymes with importance coefficient >90% and with up to 5 enzymes with highest absolute loading per component.

#### Enzyme Saturation

KM values of enzymes for *S. cerevisiae* were obtained from the BRENDA database (Chang et al., 2015, Schomburg et al., 2000) accessed via its Python API on 1.10.2015. Metabolite names were manually mapped to substrate names of BRENDA records. Since the database contains the records of multiple enzymes, including recombinant and modified proteins, only the enzymes that did not match "mutant|recombinant" pattern in the comment section were used for the analysis. For absolute concentration determination we used calibration as described previously (Mülleder et al., 2016a), i.e. by adjusting for dilution used in metabolite extraction protocols and normalising by cell volume (Figures 4F–4H). For analysis we collected 5ml cultures at OD595 1.5, the extraction volume (100 μl for Dataset 2 and 400 μl for Dataset 3) and used the values for cells/OD595 (3.2^∗^107) and cell volume (45.54 fL) for the strain BY4741 in synthetic minimal medium. Cell volume estimates were obtained from Petrezselyova et al. (2010).

#### Note on the Relationship between Enzyme Expression Changes in the High and Low Abundant Fraction of the Proteome

As microLC-SWATH-MS captures preferentially the high abundant fraction of the the proteome (Vowinckel et al., 2018) we made use of transcriptional profiles as previously recorded for the kinase deletion strains in exponentially growing cells (van Wageningen et al., 2010), in order to assess enzyme expression also within the genes of lower expression level. Enzyme encoding transcripts on average account for 15% of the total transcriptomic impact of kinase deletion (using the thresholds for differential expression as defined in van Wageningen et al. [2010]) (Figure S5).

In the subset of transcripts that directly correspond to the proteins as quantified by microLC-SWATH-MS, this value is 27%; in the subset of transcripts where no protein values are available (largely representing the low abundant fraction of the proteome), this value is 11%. Kinase-dependent enzyme level changes hence dominate to 1/3rd the highly abundant fraction of the proteome, while they are also significant, but less dominating factor, in differential gene expression in the low abundant transcript fraction.The comparison of our proteomes with this transcriptional data needs to be seen in the context that the transcriptional profiles were recorded from yeast grown in amino acid supplemented media. This fact yielded some interesting observations from the comparison on its own. Indeed, the difference between amino-acid supplemented and minimal media was reflected in a lower correlation between transcriptional and proteomic data as it is typically reported in exponentially growing yeast. This confirms our recent study revealing the importance of biosynthetic metabolism as global factor in cellular gene expression (Alam et al., 2016). Although transcriptome and proteome fold-changes correlated significantly in many of the kinase knock-outs (Pearson r > 0.25, p-value < 0.01, Figure S5), none of the Pearson correlation coefficients (PCCs) exceeded a value of >0.5 (Figure 1G); the median value, 0.12, was much lower, and in several strains the correlation was insignificant (Figures 1G and S5). Furthermore, we find that the proximity of kinases to transcription factors in protein-protein interaction networks (Szklarczyk et al., 2015) is a negative indicator of enzyme level changes (Wilcoxon rank sum test, p-value < 0.05). Hence, the more upstream a kinase is compared to a transcription factor, the more enzymes are affected (Figure S6). In contrast, the number of protein-protein interactions reported for each kinase, and the betweenness in the protein-protein interaction networks, did not show significant correlations with number of affected enzymes (Figure S6).

### Quantification and Statistical Analysis

All statistical analyses were done in R (R Core Team, 2015) with specific packages as indicated in each methods section. For the basic data manipulation and visualization we used the R tidyverse package compilation (Wickham and Grolemund, 2016). Hypothesis testing to assess means of population differences were mainly done using non-parametric Wilcoxon Rank Sum test, unless indicated otherwise in specific cases. Sample size estimation were not performed in any of the experiments. For growth experiments at least n=3 biological replicates were analysed unless stated otherwise.

### Data and Software Availability

The raw proteomics mass spectrometry data have been deposited to the ProteomeXchange Consortium via the PRIDE (Vizcaíno et al., 2016) partner repository with the dataset identifier PRIDE: PXD010529. All code used to generate figures in the manuscript are available through Github repository: https://github.com/zelezniak-lab/kinase_metabolism. All data from this manuscript is deposited at: https://doi.org/10.5281/zenodo.1320288.

## Supplemental Information

Document S1. Figures S1–S22

Document S2. Article plus Supplemental Information
