# Supplementary material for: Machine Learning Predicts the Yeast Metabolome from the Quantitative Proteome of Kinase Knockouts
Source: Cell Syst. 2018 Sep 26;7(3):269–283.e6. doi: 10.1016/j.cels.2018.08.001 (PMC6167078; doi:10.1016/j.cels.2018.08.001)
Supplement: Document S1. Figures S1–S22 [file mmc1.pdf]

Cell Systems, Volume 7

Supplemental Information

Machine Learning Predicts the Yeast Metabolome from the Quantitative Proteome of Kinase Knockouts

Aleksej Zelezniak, Jakob Vowinckel, Floriana Capuano, Christoph B. Messner, Vadim Demichev, Nicole Polowsky, Michael Mülleder, Stephan Kamrad, Bernd Klaus, Markus A. Keller, and Markus Ralser

Supplementary Information

Machine learning predicts the yeast metabolome from the quantitative proteome of kinase knock-outs

Aleksej Zelezniak[1,2,3,4], Jakob Vowinckel[2,5], Floriana Capuano[2], Christoph Messner[1], Vadim Demichev[1,2], Nicole Polowsky[2], Michael Mülleder[1,2], Stephan Kamrad[1,7], Bernd Klaus[6], Markus Keller[2,8] and Markus Ralser[1,2,9*]

[1]The Francis Crick Institute, Molecular Biology of Metabolism laboratory, London, United Kingdom

[2]Department of Biochemistry and Cambridge Systems Biology Centre, University of Cambridge, Cambridge, United Kingdom

[3]Department of Biology and Biological Engineering, Chalmers University of Technology, Gothenburg, Sweden

[4]Science for Life Laboratory, KTH – Royal Institute of Technology, Stockholm, Sweden

[5]Biognosys AG, Schlieren, Switzerland

[6]Centre for Statistical Data Analysis, European Molecular Biology Laboratory (EMBL), Heidelberg, Germany

[7]Department of Genetics, Evolution and Environment, University College London, London, United Kingdom

[8]Medical University of Innsbruck, Innsbruck, Austria

[9]Department of Biochemistry, Charité Universitaetsmedizin Berlin, Berlin, Germany

*Lead author. +44 1223 761346, markus.ralser@crick.ac.uk

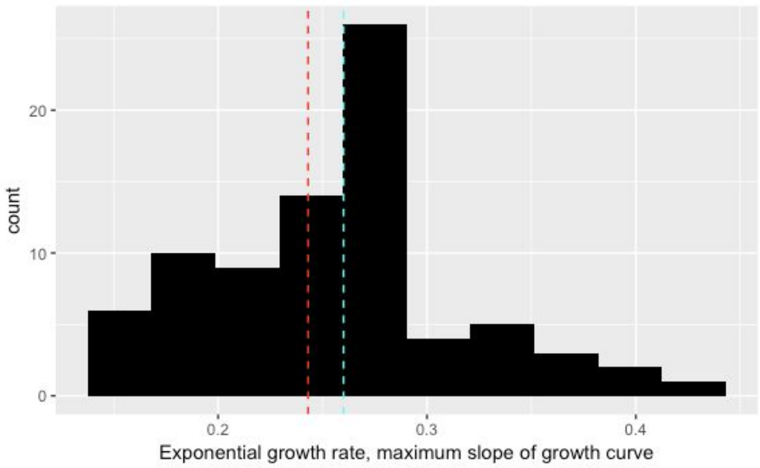

Figure S1. Related to Figure 1; Growth rate in kinase mutants. Many kinase mutants (median growth rate of all kinases, cyan dotted line) exhibit growth rates similar to WT (red dotted line). Data is non-normally distributed with mass center close to WT-strain. The growth curves were fitted using non-parametric (without growth law assumption) spline model as implemented in R growFit package (Kahm et al., 2010).

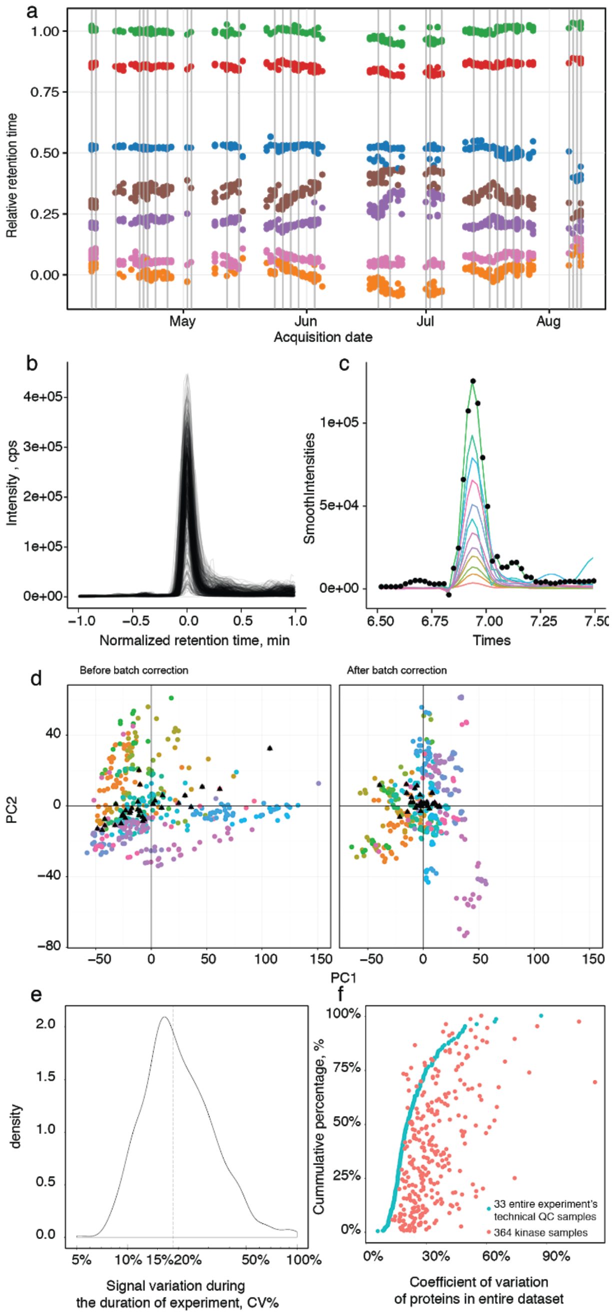

Figure S2. Related to Figure 1; Quality of large proteomics experiment a)microLC-SWATH-MS (Vowinckel et al., 2018) was applied to systematically record the proteomes of *Saccharomyces cerevisiae kinase gene* deletion strains. Shown are retention time stabilities during the measurement of 397 yeast full-proteome tryptic digests by microLC-SWATH-MS over a four-month acquisition period. The median retention time drift was as low as +/- 5.7%, as illustrated by the retention of standard peptides (iRT, coloured points). The rightmost coloured dots represent average peptide retention time with standard deviation (in % of iRT retention) of total chromatographic runtime. Grey lines indicate the processing of a standardised proteome digest (quality control (QC) sample) to monitor instrument performance, to normalise for batch effects, as well as to determine adequate cut-off values for determining differential protein expression. **b)** Overlay of 397 extracted ion chromatograms representing a typical iRT peptide (IGSEVYHNLK) illustrates chromatographic robustness. **c)** our microLC-SWATH-MS implementation covered the typical chromatographic peak with a 1.31s scan cycle so that the illustrated example peptide IGSEVYHNLK (left) is covered by 9 MS^2 and 3 MS^1 ions (different colours in the chromatogram), each by 10 measurements (black dots) in the average sample. This high coverage helps to obtain precise quantification. **d**) Batch correction of microLC-SWATH-MS proteomic data. Before batch correction signal is technically confounded by the acquisition date as demonstrated by variation of external QC control samples (black triangles), colours represent different experimental batches acquired in the period of 4 months. Batch correction reduces variation associated with acquisition date as demonstrated by grouping of QC samples (right panel). **e)** Technical variation of label-free protein quantification as determined by calculating coefficient of variation of combined fragment signal batch corrected intensities in all quality control samples. x - axis is log-scaled, dotted line is the median of the CV% values (19%). **f)** The coefficient of variation (CV) at whole-process technical and biological levels. The CV of technical replicates in 93% of measured metabolic enzymes were lower than in kinase samples, resolving biological signal from technical noise. Kinase samples are sorted as QC replicates.

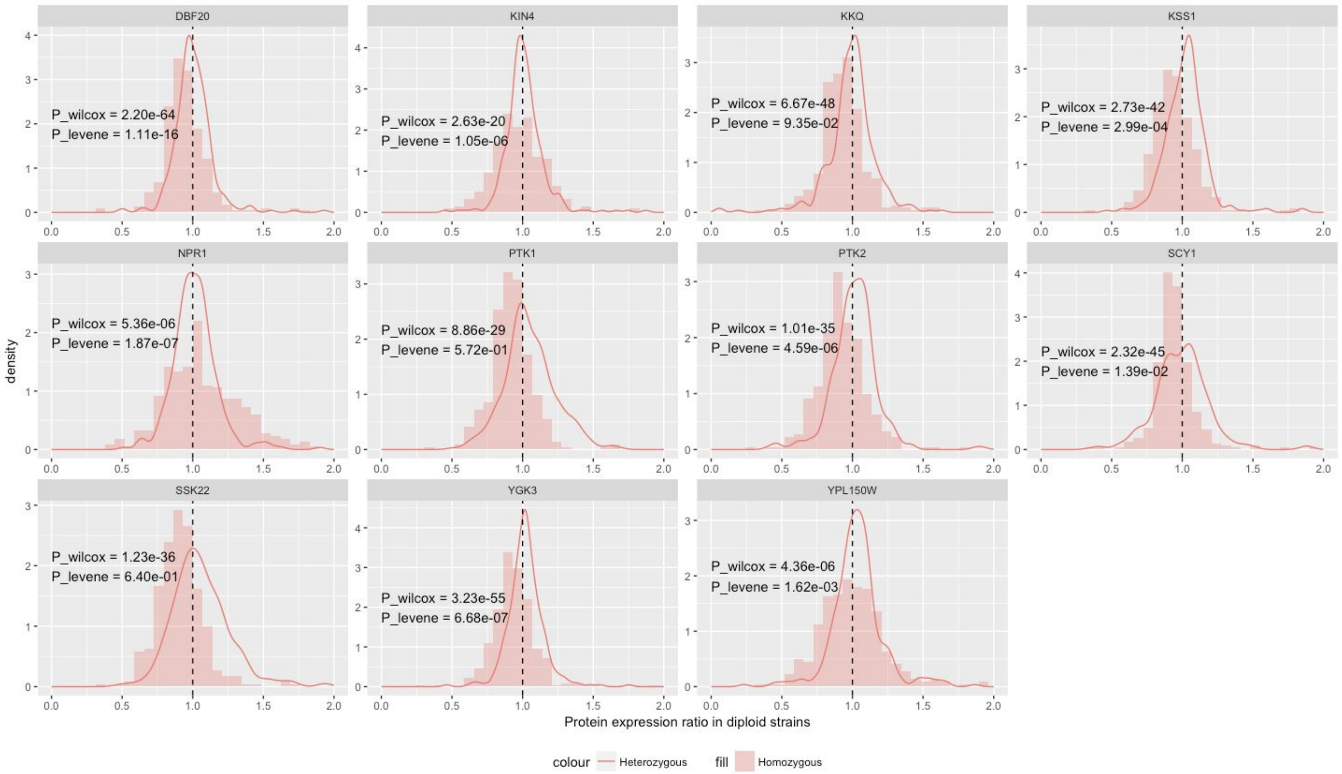

Figure S3. **Related to Figure 1; Kinase deletions in diploids.** Enzyme expression in ten heterozygous vs homozygous kinase mutants, generated by mating the *MATa* strains as used in our study (Mülleder et al., 2012) with a wild-type strain (BY4742) or a complementary kinase knock-out in the MATalpha background. Homozygous diploid kinase mutants have much stronger gene expression changes compared to the wild-type, relative to the corresponding heterozygous strains to which one kinase copy was re-introduced by mating with the MATalpha kinase-wild-type strain. Histogram represents a ratio between kinase homozygous diploid mutant and diploid BY4741-*Δhis* parental strain. Density plot shows ratio between heterozygous mutants normalized by their respective MATa/*MAT*α-*Δkinase* vs *MATa*/*MAT*α-WT diploids*.* The dotted line corresponds to no change respective to the wild-type control proteome.

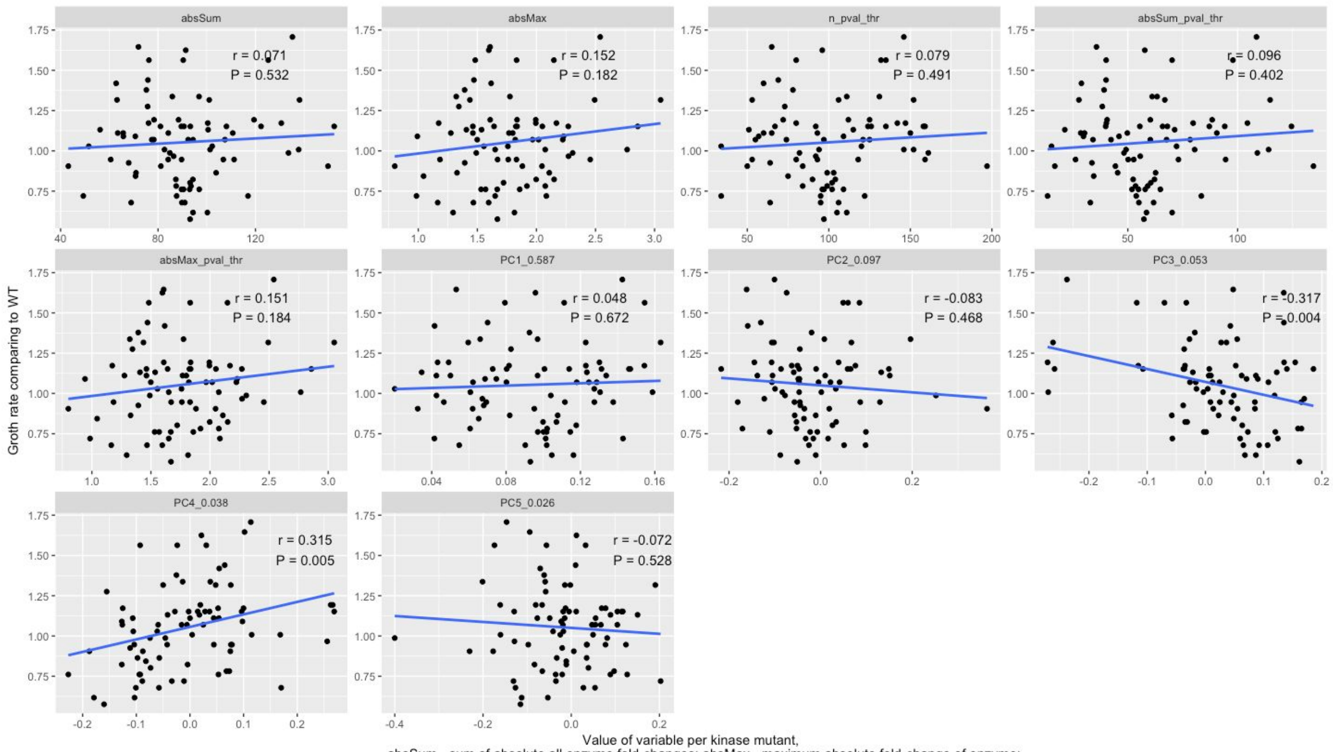

absSum - sum of absolute all enzyme fold-changes; absMax - maximum absolute fold-change of enzyme;
n_pval_thr - number of differentially expressed enzymes using BH adjusted p-value cutoff;
absSum_pval_thr - sum of absolute differentially expressed enzymes fold-changes using BH adjusted p-value cutoff;
absMax_pval_thr - maximum absolute fold-change of differentiallty expressed enzyme using BH adjusted p-value cutoff;
PCX_YYY - scores of principal component X based on fold-changes of all enzymes, YYY is the fraction of total variance explained by the principal component.

Figure S4. Related to Figure 1; Changes in growth rate are not the main cause of differential enzyme expression in kinase knock-outs, as the main principal components of enzyme expression show no correlation with growth rate changes. Such correlation is however obtained between principal components 3 and 4, that capture 5.3% and 3.8% respectively, of total enzyme expression. More than 90% of enzyme expression changes in kinase knock-outs are not associated to growth rate changes.

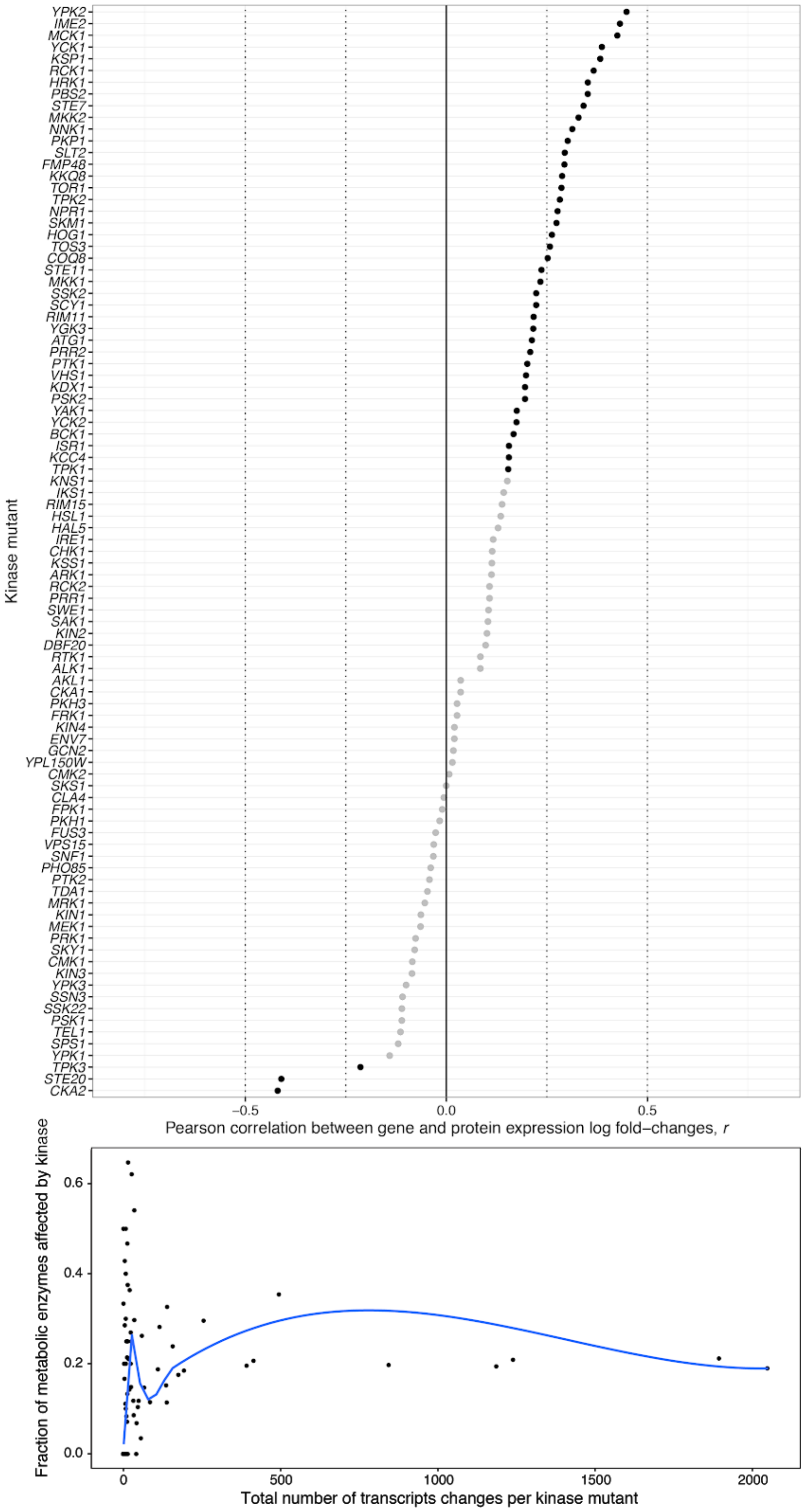

Figure S5. **Related to Figure 1; Correlation between mRNA and protein expression in kinase mutants.** Top: Correlation between mRNA and protein expression $\log_2$ fold-changes in kinase knock-outs. Grey points represent correlation coefficients where p-value exceeded significance cutoff >0.01. Bottom: Fraction of differentially expressed enzymes-coding genes, in comparison to all

differentially expressed genes, at the transcriptional level in kinase deletion strains (van Wageningen et al., 2010). Multiple kinase transcriptomes are characterized by a high number of differentially expressed enzymes, 16% on average. As on the proteome, the total effect size does not determine the relative occurrence of enzyme-encoding transcripts. Fold-change and p-value cutoffs for differential gene expression were obtained from the the original publication (van Wageningen et al., 2010).

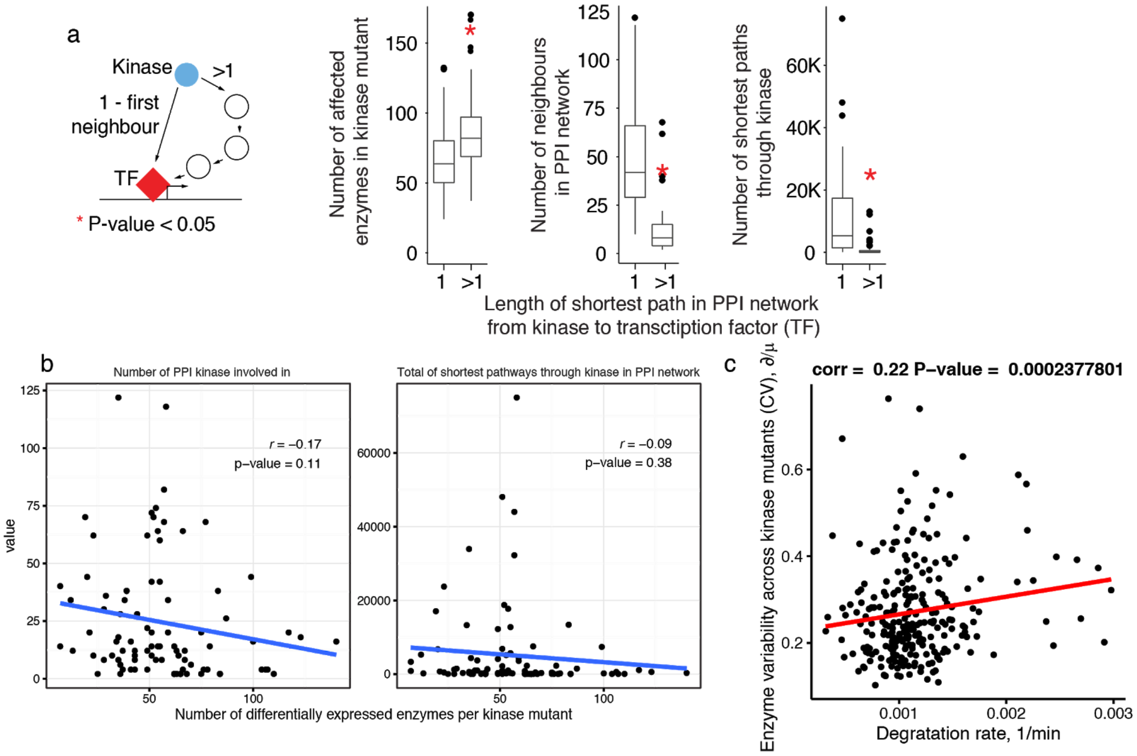

Figure S6. Related to Figure 1; a) The more distant a kinase is to a transcription factor (TF) in a protein-protein interaction network, the more enzyme levels it affects (Wilcoxon rank sum test, p-value < 0.05) (left). Conversely, kinases which directly interact with a transcription factor, have a higher network centrality (middle) and an increased betweenness (right). However, their importance in PPI network had no influence on the number of differentially expressed enzymes (b). **b)** Perturbation size, expressed as a number of differentially expressed enzymes in contrast to wild type strain, is not correlated with number of protein-protein interactions (PPI) kinase involved in (left panel) or number of shortest paths going through kinase in PPI networks kinase (right panel). **c)** Protein degradation rate (x-axis) and the likelihood of an enzyme to be regulated by a kinase, expressed as coefficient of variation across all kinases mutants (y-axis) are weakly correlated. Enzyme degradation rates were obtained from (Christiano et al., 2014)**.** For network analysis a collection of yeast protein-protein interactions (PPI) was obtained from the STRING database (Szklarczyk et al., 2015) (version 10, downloaded on 2015-06-03). We constructed a high confidence (STRING score > 900) PPI network based only on experimentally validated interactions. Transcription factor annotation were obtained based on GO slim (www.yeastgenome.org) categories by selecting terms matching the “nucleic acid binding transcription factor activity” pattern. Graph manipulations and network analysis were performed using the *igraph* library as implemented in R package (Csardi and Nepusz, 2006).

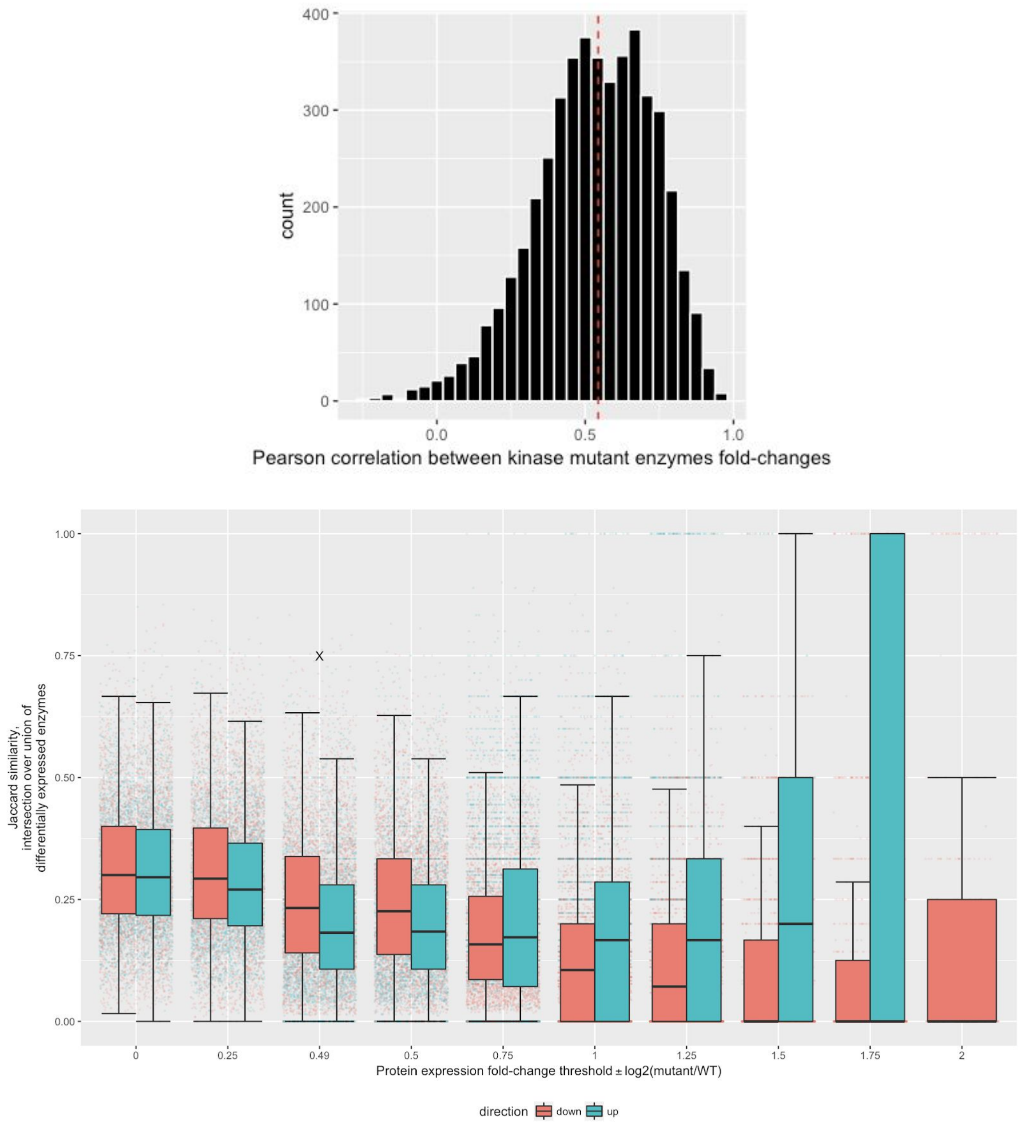

Figure S7. Related to Figure 2; Sensitivity analysis of differential expression. Top panel: Distribution of correlations of kinase mutant enzyme fold-changes. The median correlation of between kinase signatures is ~0.5. A simple linear model build on this basis shows that only 25% of expression changes of one kinase can explain changes of the other, leaving ¾ of the proteome changes being specific to the typical kinase deletion. Hence, also with this metric, the conclusions holds: each kinase deletion leaves a highly specific signature in the enzyme expression proteome. Bottom panel: Sensitivity analysis applied to protein differential expression cutoffs in kinase knock-out strains. Symbol "X" denotes the threshold applied in our study. As one can observe similarity of differentially expressed genes is low between kinase knock-out proteomes even when consider no, or conservative, fold-change cutoff. Dots on the background represent Jaccard similarity of all pairwise kinase comparisons of differentially expressed enzymes. Please note, that as typical for enzyme expression experiments, with there are very few genes that have very strong fold-change concentration changes, thus dots are not anymore gradually scattered once large thresholds are applied.

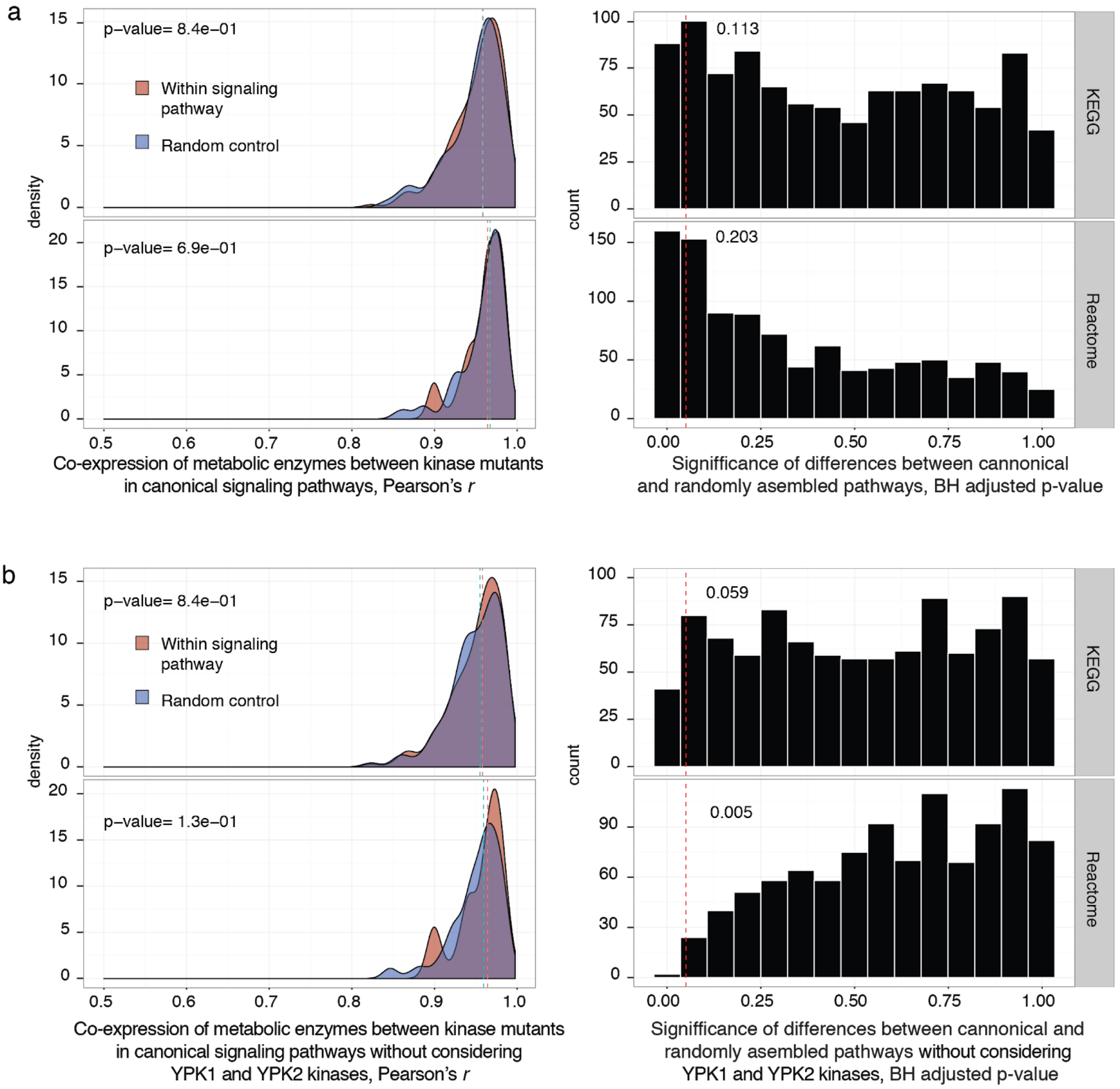

Figure S8. Related to Figure 2; Kinase mutants expression mapping to signaling pathways (part 1). Kinase signaling pathways as assembled in KEGG and REACTOME, and the kinase associations within them, fail to explain enzyme co-expression upon kinase deletion. a) The distribution of the correlation coefficients between enzyme expression levels in kinases mutants that are annotated to the same signaling pathway from KEGG or Reactome databases (left panel). The distribution corresponding to random assignment (of kinases to signaling pathways of the same size as the annotated signaling pathways) is shown for comparison. Random pathways and signaling pathways predict enzyme expression changes not statistically different. Right panel, distribution of p-values from tests (Wilcoxon rank sum) comparing co-expression of canonical signalling versus 1000 times randomly assigned pathways of the same size. Dotted red vertical line denotes a fraction of significantly detected differences (BH adjusted p-value <0.05) between coexpression in canonical pathways and random background. b) same as in (a), but removing *YPK1/ YPK2* - the kinase pair that is most frequently annotated to signaling kinases. In total 49 kinases were assigned to signaling pathways in both databases.

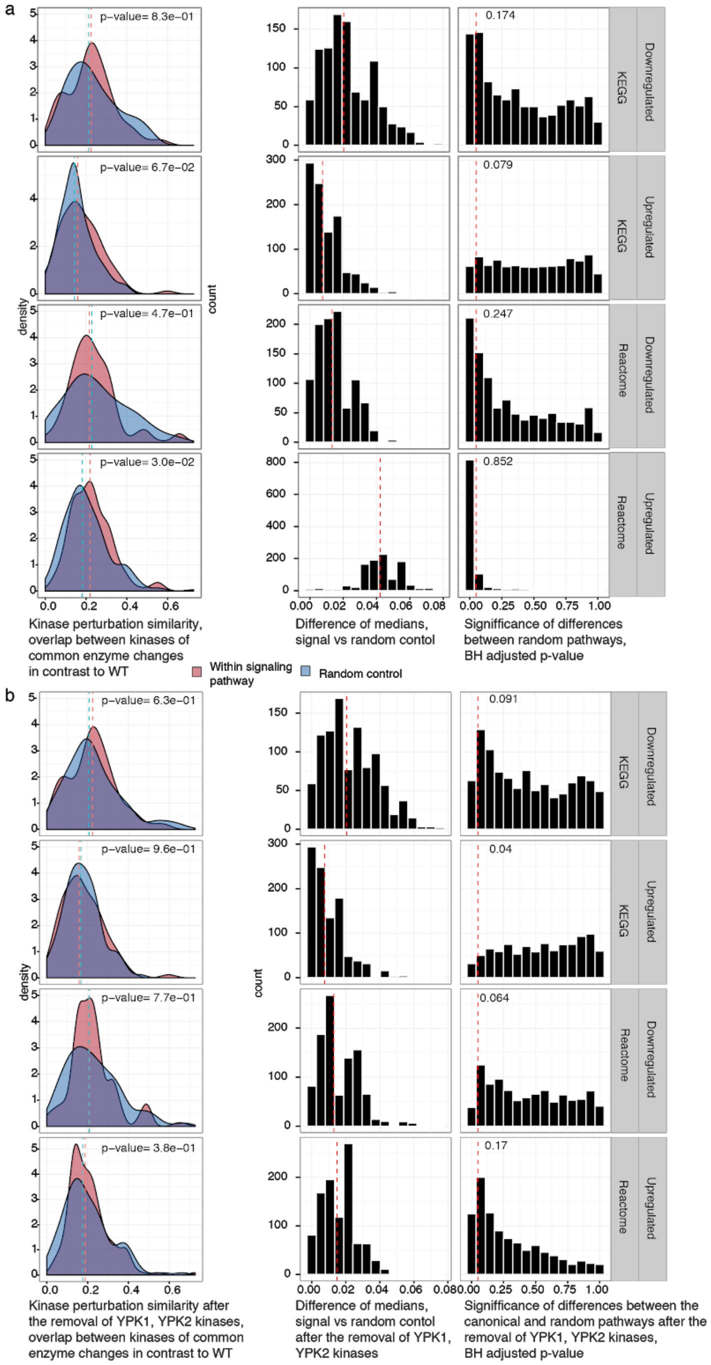

Figure S9. Related to Figure 2; Kinase mutants expression mapping to signaling pathways (part 2). The conventional topology of signaling pathways, and the kinase associations within them, fail to explain enzyme expression upon kinase deletion. The distribution of the overlaps in up-/downregulated metabolic enzymes levels (>|$\log_2$(fold-change)|, BH adj. p-value < 0.01 in contrast to WT strains, see STAR Methods) in kinases mutants that are annotated to the same signaling pathway from KEGG or Reactome databases (left panel). The distribution corresponding to random assignment of kinases to signaling pathways of the same size is shown for comparison. [Middle panel] distribution of median differences of overlaps between canonical kinase and randomly assembled pathways of the same size. Right panel, distribution of p-values from tests (Wilcoxon rank sum) comparing overlaps in canonical signalling versus 1000 times randomly assigned pathways of the same size. Dotted red vertical line

denotes a fraction of significantly detected differences (BH adjusted p-value <0.05) between overlaps in canonical pathways and random background. b) same as in (a), but removing *YPK1, YPK2* - the kinase pair that is most frequently annotated to signaling kinases. In total 49 kinases were assigned to signaling pathways in both databases.

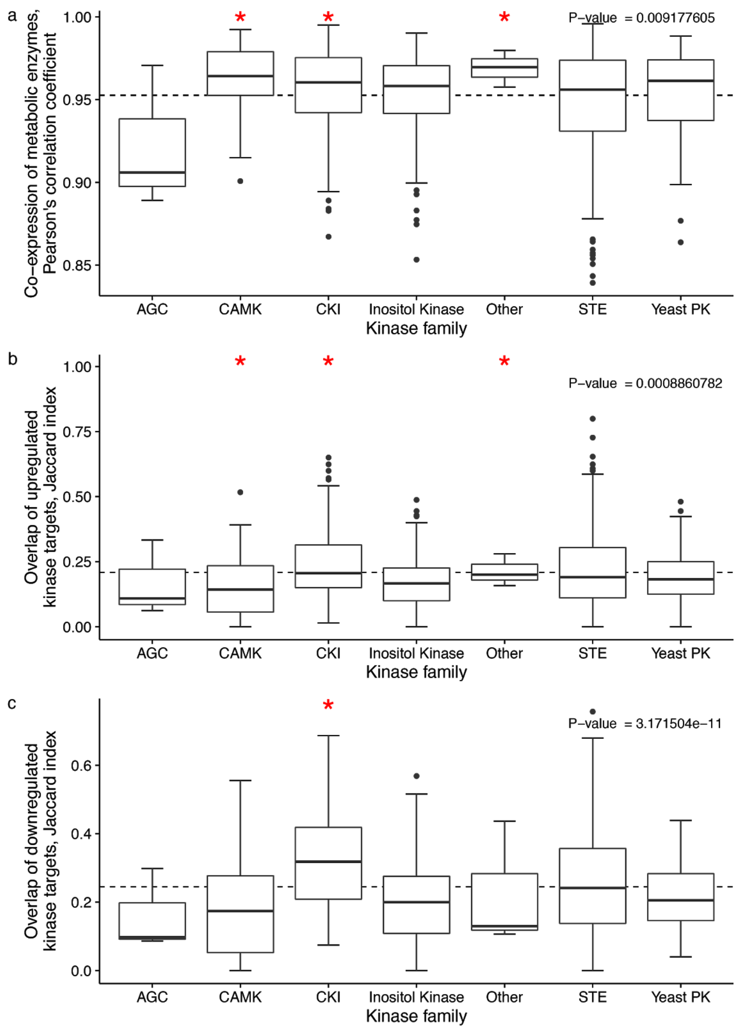

Figure S10. **Related to Figure 2; Co-expression of enzymes between kinase families.** Definition of kinase classes was taken from (Hunter and Plowman, 1997). a) Co-expression of metabolic enzymes between kinase within the class of kinases, expressed as Pearson's correlation coefficient. b,c) overlaps of up-/downregulated metabolic enzymes in kinase mutants. P-values denote significance of one-way ANOVA test using kinase family (classes) as categorical variable, stars depict variables that are significantly different from mean response levels (dashed line).

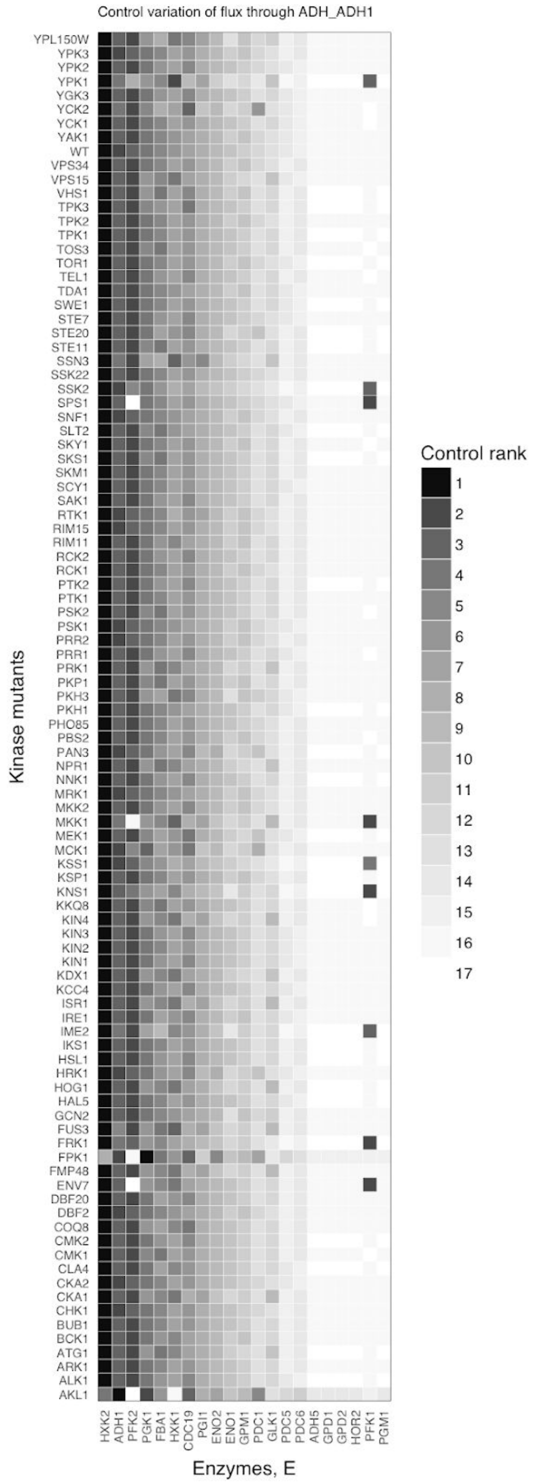

Figure S11. **Related to Figure 3; Flux control variation over alcohol dehydrogenase (*ADH1*) in kinase knockouts.** Control of flux through alcohol dehydrogenase (*ADH1*) reaction shifts to other enzymes depending on the enzymes expression in each kinase mutant. Control coefficients are ranked with the highest as rank 1.

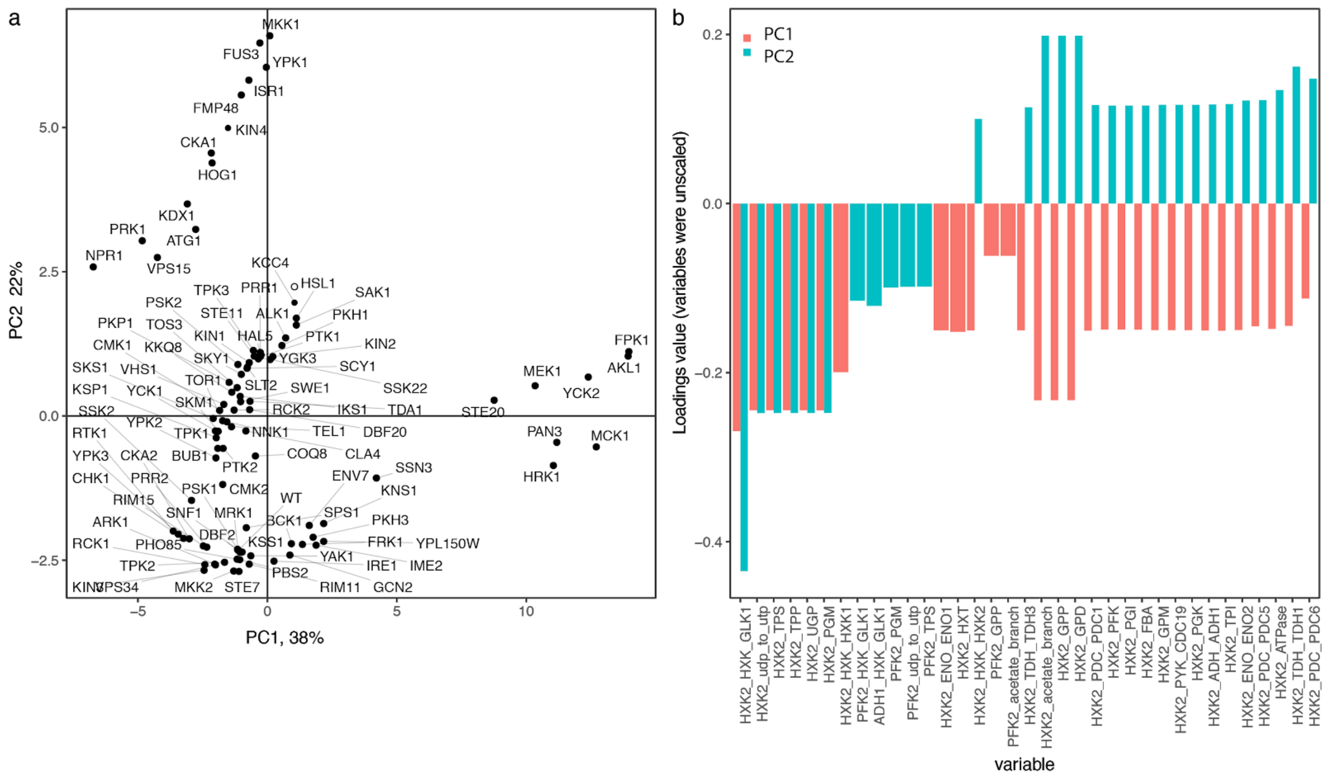

Figure S12. **Related to Figure 3; Flux control variation in kinase mutants using principal component analysis.** a) Principal component plot of flux control coefficients (FCC) for every kinase mutant. FCC were not scaled. Values on axes labels represent percentage of total variance explained by each of the component. b) Loadings for 2 principal components, for each component top 30 (absolute values) FCC are plotted colored according to the component variable loads on.

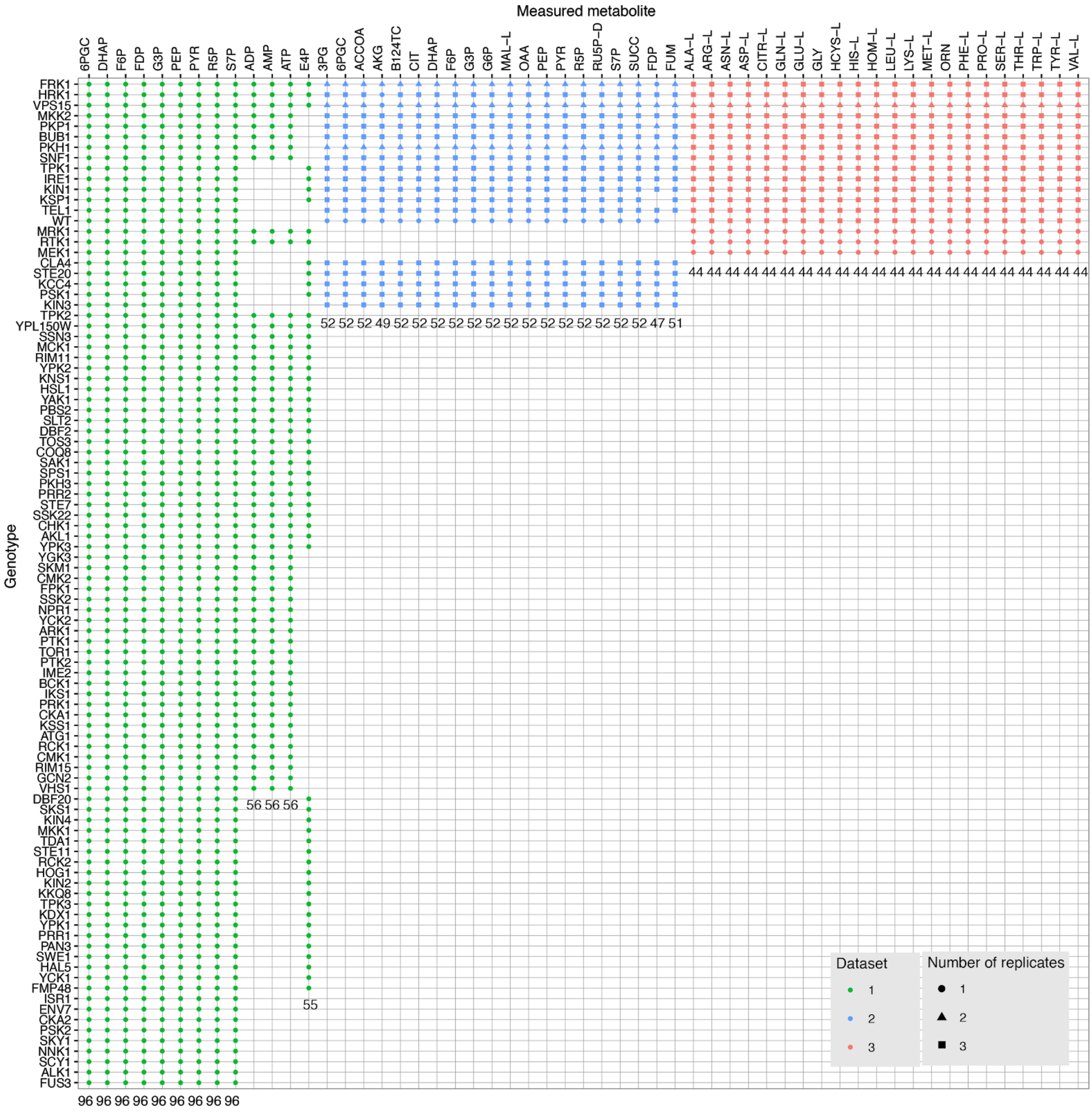

Figure S13. Related to Figure 3, Figure 4, Figure 5; Overview over the metabolite datasets used in the study. Numbers displayed below of each metabolite indicate the total of samples where metabolite was measured.

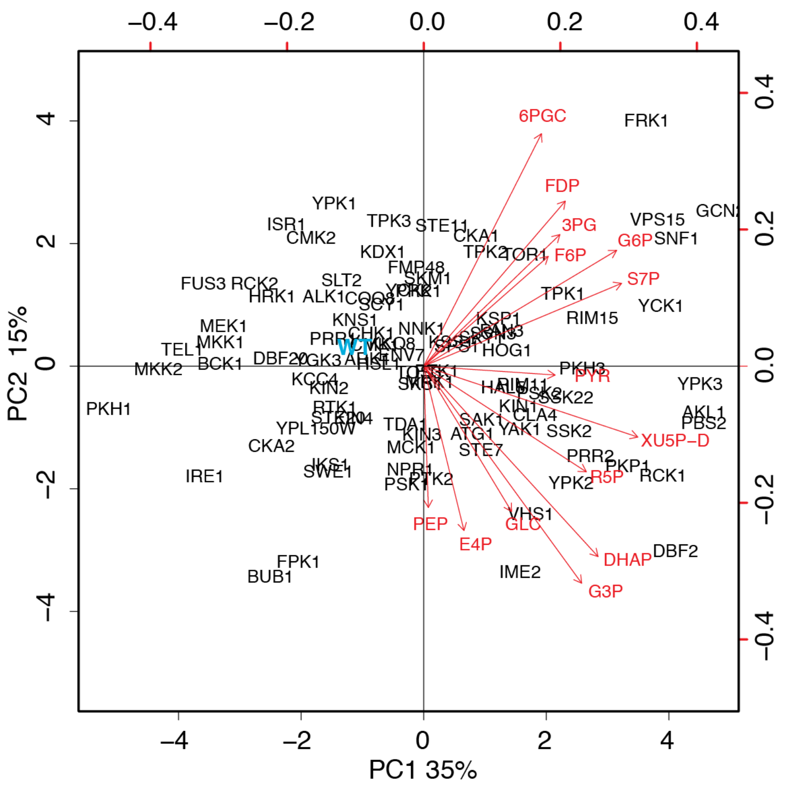

Figure S14. **Related to Figure 3, Figure 4, Figure 5; Principal component analysis of the metabolic profiles caused by kinase deletion.** Percentages on the left and bottom axes denote the proportion of the total metabolite concentration variance captured by the first two principal components. The arrows denote the contribution of each metabolite towards the principal component (loading plot). In blue is highlighted wild-type strain. For visualization purposes only, missing concentration values were imputed as described in (Honaker et al., 2011). Metabolite abbreviations: FDP Fructose 1,6 bisphosphate, 6PGC: 6 -phosphogluconate, FDP: fructose 1,6-bisphosphate, 3PG: 3-phosphoglycerate, F6P: fructose 6-phosphate, G6P: glucose 6-phosphate, S7P: sedoheptulose 7-phosphate, PYR: pyruvate, XU5P-D: xylulose 5-phosphate, R5P: ribose 5-phosphate, DHAP: Dihydroxyacetone phosphate, G3P: Glyceraldehyde 3-phosphate, GLC: glucose, E4P: erythrose 4-phosphate, PEP: phosphoenolpyruvate.

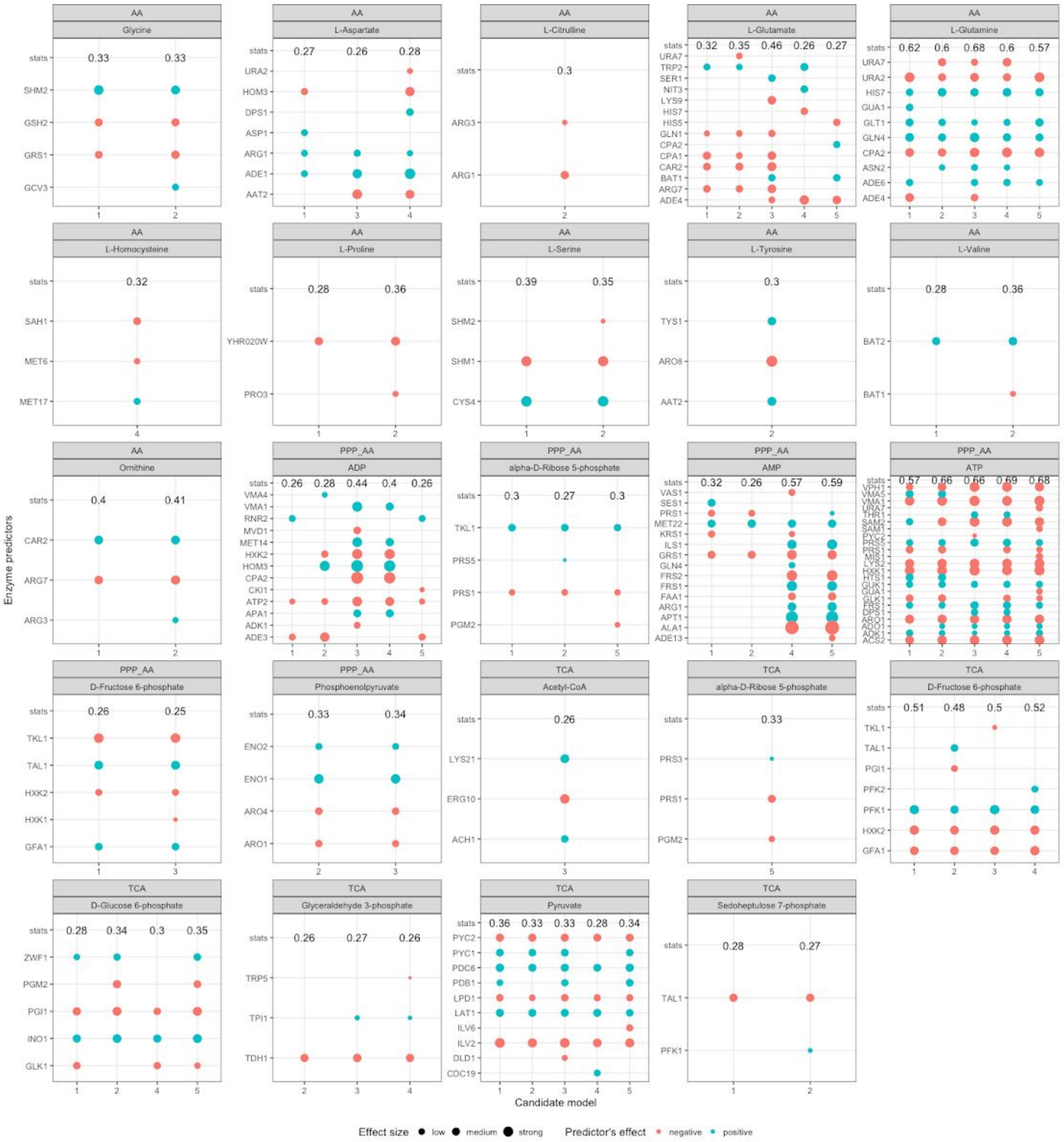

Figure S15. Related to Figure 4; Metabolite concencentration models formulated using multiple linear regression model with exhaustive feature selection. Stats - represents adjusted R^2, all models were diagnosed for the presence of autocorrelation, outliers and influential points (Methods). Presented models have adjusted R^2 value >0.25 and p.value < 0.01. In the main text the models with highest adj. R^2 are presented. For ATP metabolite, due to its connectivity in metabolic network the number of explanatory variables was exceeding the number measured samples, thus before feature selection the explanatory

variables were transformed onto principal components to reduce the dimensionality. From each component we chose 2 highest absolute loadings and assigned corresponding regression coefficient from selected feature.

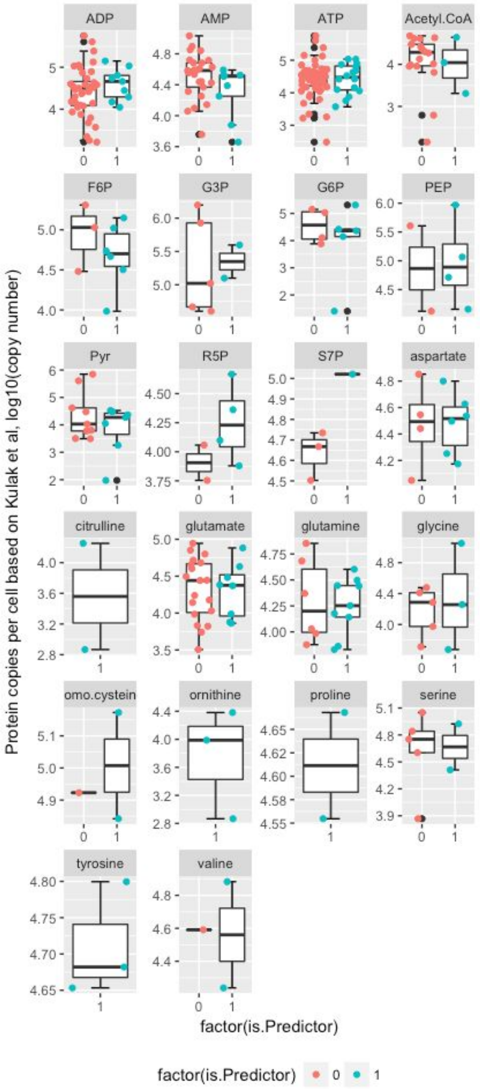

Figure S16. Related to Figure 4; No difference in absolute copy numbers between the best explanatory variables of metabolite concentrations and the rest enzymes. The best predictors were selected by exhaustive feature selection using multiple linear regression. For tyrosine, homo-cysteine and ornithine the only measured directly metabolizing enzymes are the ones which are displayed and therefore solely identified as predictors.

	Algorithm	RMSE	RsquaredCV	Data transformation	Dataset	Metabolite	Pathway	Network radius
1	enetModel	0.72	0.58	Box–Cox	AA	L–Arginine	Glutamate family	2
2	plsModel	0.61	0.67	Box–Cox	AA	L–Aspartate	Aspartate family	2
3	plsModel	0.60	0.66	Box–Cox	AA	Glycine	Serine family	3
4	enetModel	0.63	0.59	Box–Cox	AA	L–Histidine	His&nucleotide	2
5	plsModel	0.73	0.57	Box–Cox	AA	L–Homoserine	Other	2
6	fobaModel	0.66	0.65	log	AA	L–Alanine	Pyruvate family	3
7	gbmModel	0.73	0.51	log	AA	L–Asparagine	Aspartate family	2
8	fobaModel	0.77	0.55	log	AA	L–Glutamate	Glutamate family	3
9	plsModel	0.56	0.72	log	AA	L–Phenylalanine	Aromatic family	3
10	plsModel	0.62	0.73	log Quantile	AA	L–Citrulline	Other	3
11	fobaModel	0.75	0.55	log Quantile	AA	L–Glutamine	Glutamate family	1
12	earthModel	0.81	0.47	log Quantile	AA	L–Homocysteine	Other	2
13	fobaModel	0.69	0.58	log Quantile	AA	L–Leucine	Pyruvate family	2
14	rpartModel	0.87	0.44	log Quantile	AA	L–Methionine	Serine family	1
15	gbmModel	0.59	0.75	log Quantile	AA	Ornithine	Other	3
16	plsModel	0.71	0.60	log Quantile	AA	L–Valine	Pyruvate family	3
17	earthModel	0.75	0.54	log	AA	L–Serine	Serine family	3
18	fobaModel	0.54	0.72	Box–Cox	AA	L–Lysine	Glutamate family	2
19	rpartModel	0.80	0.51	Box–Cox	AA	L–Proline	Glutamate family	2
20	plsModel	0.65	0.60	Box–Cox	AA	L–Threonine	Aspartate family	3
21	fobaModel	0.52	0.75	Box–Cox	AA	L–Tryptophan	Aromatic family	2
22	svmRModel	0.77	0.52	Box–Cox	AA	L–Tyrosine	Aromatic family	1
23	enetModel	0.82	0.41	Box–Cox	PPP_AA	ADP	Energy metabolism	3
24	fobaModel	0.78	0.46	Box–Cox	PPP_AA	AMP	Energy metabolism	3
25	fobaModel	0.74	0.55	Box–Cox	PPP_AA	Erythrose 4–phosphate	PPP	2
26	enetModel	0.90	0.31	log Quantile	PPP_AA	ATP	Energy metabolism	1
27	svmRModel	0.88	0.34	Box–Cox	TCA	cis–Aconitate	TCA	3
28	fobaModel	0.79	0.50	Box–Cox	TCA	D–Fructose 6–phosphate	Glycolysis	2
29	plsModel	0.78	0.54	Box–Cox	TCA	Fumarate	TCA	3
30	svmRModel	0.78	0.52	log	TCA	Acetyl–CoA	TCA	2
31	fobaModel	0.86	0.39	log	TCA	Dihydroxyacetone phosphate	Glycolysis	2
32	rpartModel	0.80	0.54	log	TCA	D–Glucose 6–phosphate	Glycolysis	2
33	svmRModel	0.75	0.48	log Quantile	TCA	2–Oxoglutarate	TCA	1
34	gbmModel	0.63	0.53	log Quantile	TCA	Citrate	TCA	2
35	plsModel	0.72	0.56	log Quantile	TCA	Glyceraldehyde 3–phosphate	Glycolysis	3
36	fobaModel	0.78	0.46	log Quantile	TCA	Oxaloacetate	TCA	2
37	fobaModel	0.72	0.58	log Quantile	TCA	D–Ribulose 5–phosphate	PPP	3
38	fobaModel	0.87	0.43	log Quantile	TCA	Succinate	TCA	2
39	rpartModel	0.81	0.57	log Quantile	TCA	3–Phospho–D–glycerate	Glycolysis	3
40	earthModel	0.67	0.65	log	TCA	alpha–D–Ribose 5–phosphate	PPP	3
41	gbmModel	0.72	0.55	log	TCA	Sedoheptulose 7–phosphate	PPP	2
42	earthModel	0.81	0.40	Box–Cox	TCA	L–Malate	TCA	3
43	fobaModel	0.58	0.69	Box–Cox	TCA	Phosphoenolpyruvate	Glycolysis	3
44	gbmModel	0.80	0.48	Box–Cox	TCA	Pyruvate	Glycolysis	2
45	plsModel	0.73	0.53	Box–Cox	TCA	D–Fructose 1,6–bisphosphate	Glycolysis	3
46	enetModel	0.70	0.56	Box–Cox	TCA	6–Phospho–D–gluconate	PPP	3

Figure S17. Related to Figure 5; Best performing ML algorithms for metabolite concentration predictions. Metabolite data was Box-Cox transformed using the maximum log-likelihood method for parameter estimation. RsquaredCV - 100 times repeated 10-fold cross-validated R^2. Algorithms abbreviations: enetModel - Elastic net regression, plsModel - partial least squares regression, fobaModel - ridge regression with variable selection, earthmodel - multivariate adaptive regression splines, svmRModel - support vector machine regression, Hyperparameter tuning grid ranges for each algorithm are deposited at github https://github.com/alzel/regression_models/blob/master/regression_models.R

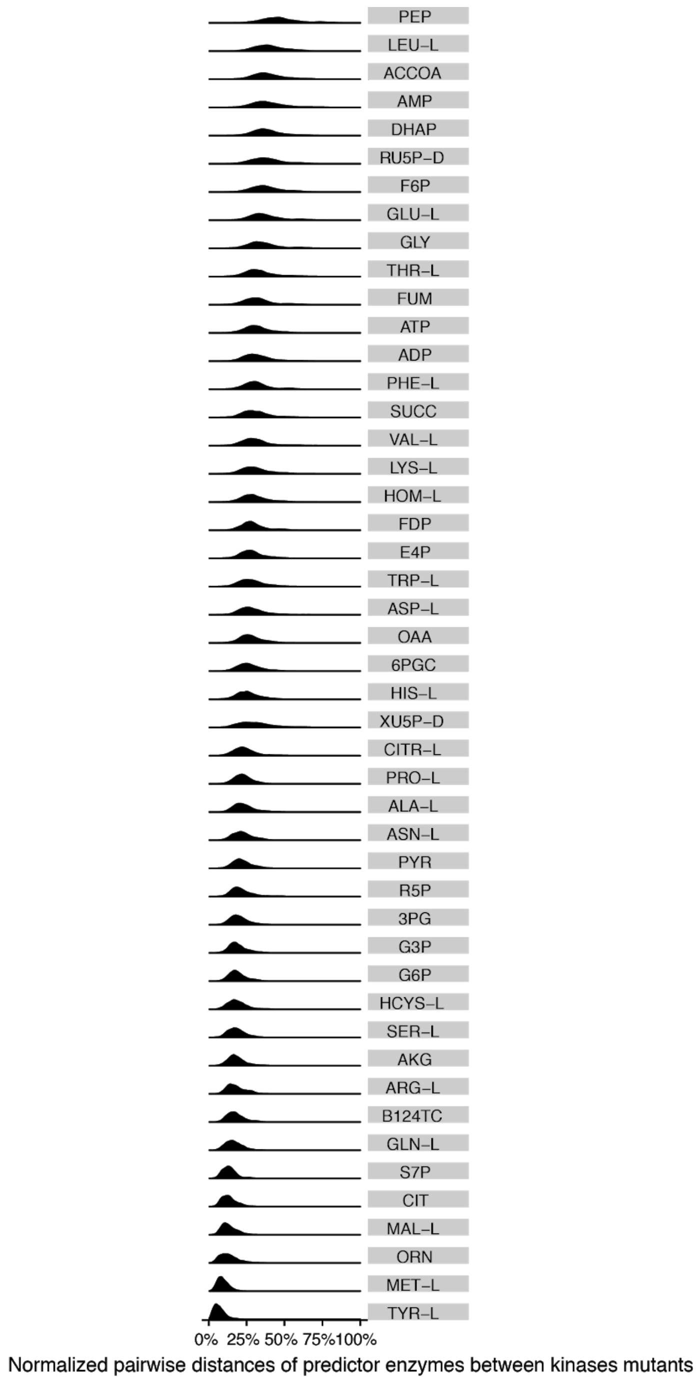

Figure S18. **Related to Figure 5; Regulatory specificity of enzyme predictors.** Kinases interact with metabolite concentrations with different degree of specificity, illustrated as response similarity distribution for each metabolite. More distant values (upper density plots) imply specific response in metabolite predictors. To compare predictor responses between metabolites, predictors were standardized (to mean zero and unit variance), and then the Euclidean distance of standardized enzyme expression was computed pairwise between each kinase mutant and normalized to 100% by the most distant kinase pair.

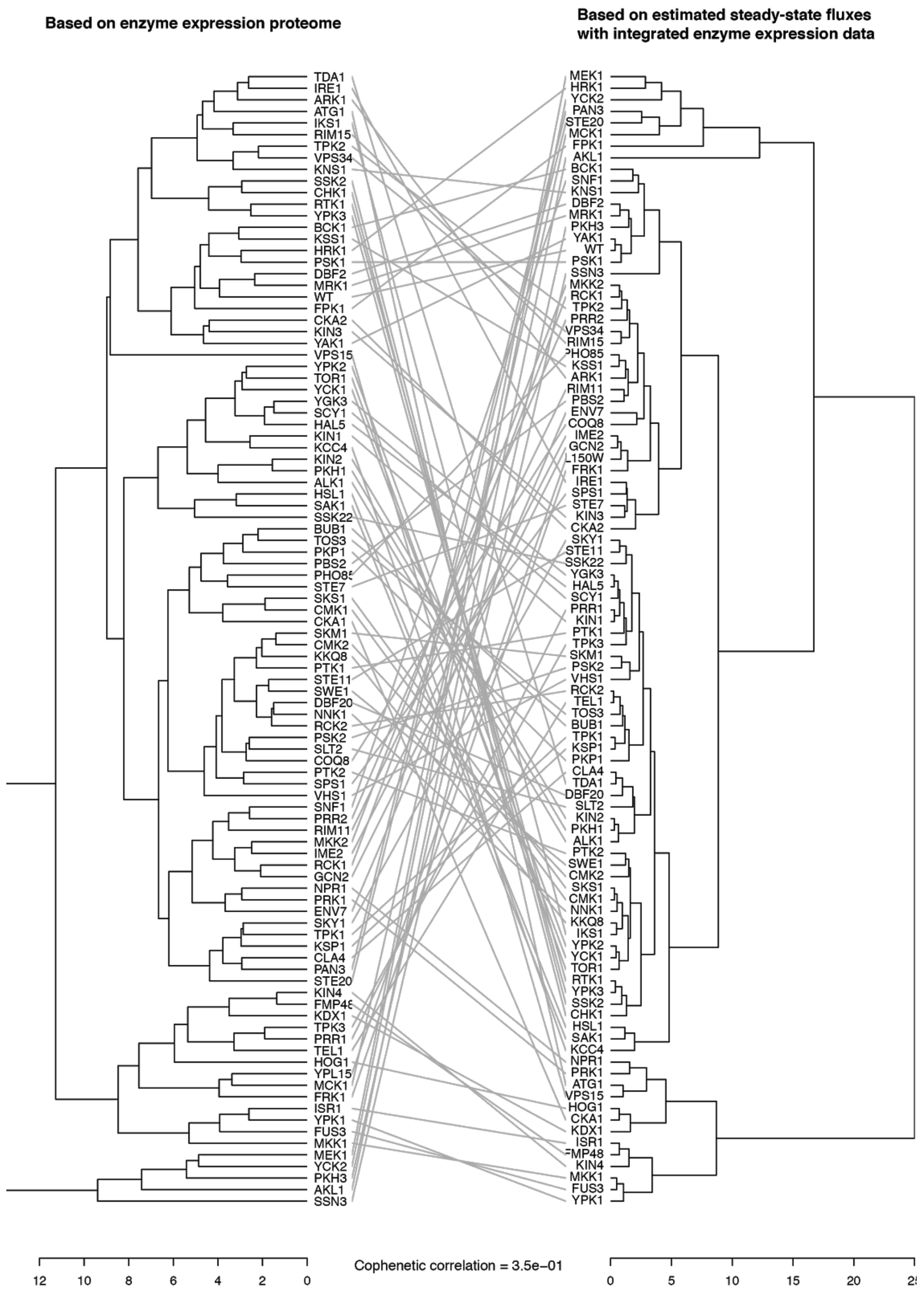

Figure S19. **Related to Figure 6; Correlation between enzyme expression and metabolic fluxes.** The nonlinear nature of metabolism regulation as highlighted by a low correlation of enzyme expression and fluxes - that were estimated by upon introducing experimentally measured enzyme abundances change into a quantitative model of glycolysis (Smallbone et al., 2013). Hierarchical clustering of kinase mutants on the basis the enzyme expression levels (left panel) and mutant fluxes calculated using same enzyme abundance for modelling (right panel). Each variable, either flux and proteins MS signal levels, were standardized by subtracting mean of the value and dividing by its standard deviation among all mutants. Using Euclidean distance between strain profiles both matrices then were hierarchically clustered with complete linkage agglomeration. Replicates of proteomics measurements were averaged per genotype and were used for both analyses.

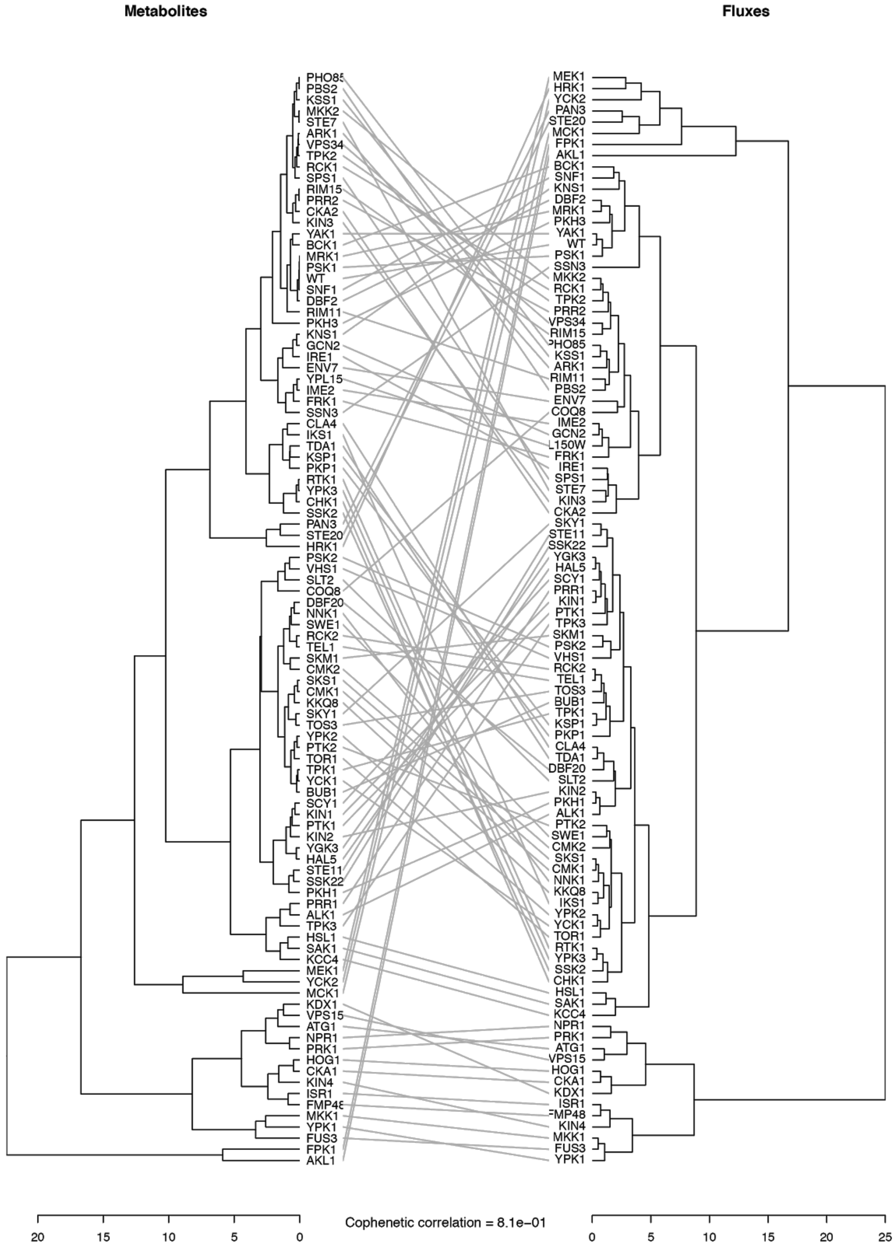

Figure S20. Related to Figure 6; Correlation between metabolite concentrations and metabolic fluxes. Metabolite concentrations are highly correlated with metabolic fluxes highlighting stronger dependency of the flux on metabolite levels rather than enzymes in kinetic model of central carbon metabolism. Analogous results were reported in several recent recent studies (Hackett et al., 2016; Millard et al., 2017). Hierarchical clustering of kinase mutants on the basis of modeled metabolite concentrations with incorporated enzyme expression levels (left panel) and mutant fluxes calculated using same enzyme abundance for modelling (right panel). Each variable, either flux and proteins MS signal levels, were standardized by subtracting mean of the value and dividing by its standard deviation among all mutants. Using Euclidean distance between strain profiles both matrices then were hierarchically clustered with complete linkage agglomeration. Replicates of proteomics measurements were averaged per genotype and were used for both analyses.

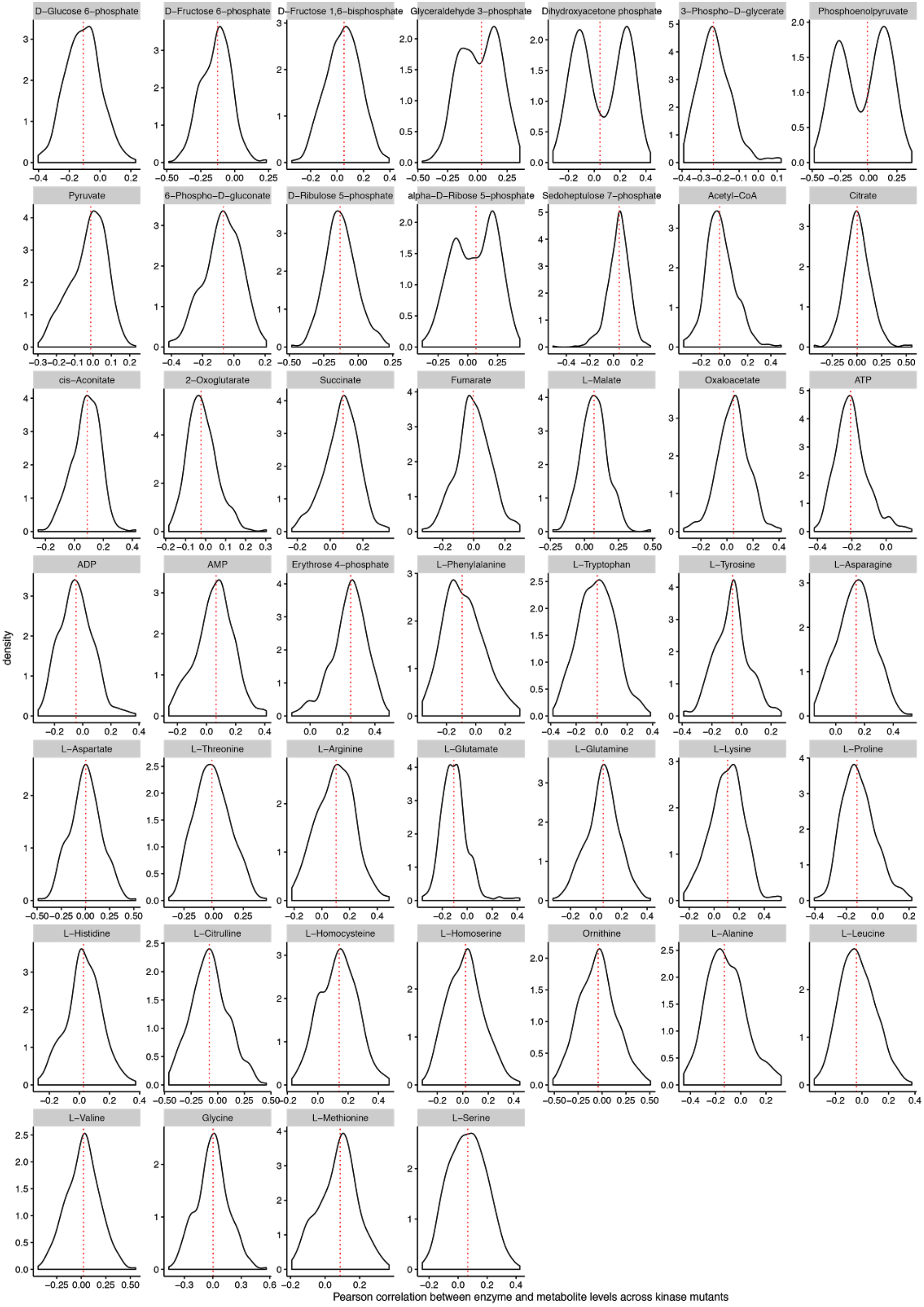

Figure S21. Related to Figure 6; Correlation between metabolite levels and enzyme abundances. Distribution of Pearson's correlation coefficients between metabolite levels and all measured enzyme abundances across all kinase mutants.

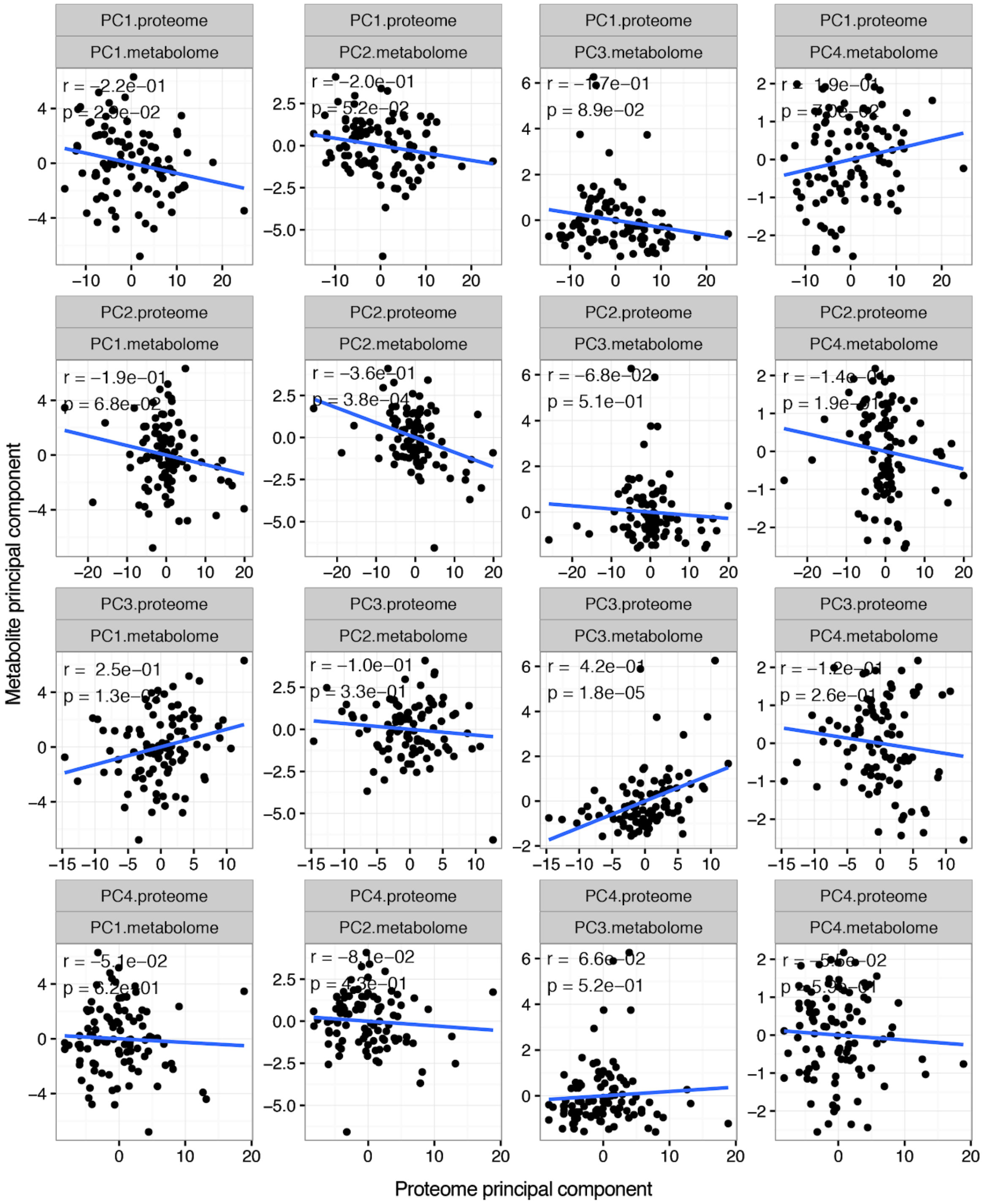

Figure S22. Related to Figure 6; Correlation of first four principal components of metabolite and proteome data. Before performing principal component analysis, each dataset was scaled, mean centered and normalized to unit variance. Metabolite data from dataset 1 (Supplementary Figure 20) was used for analysis. Missing metabolite measurements were imputed using *amelia* (Honaker et al., 2011). Replicates of proteomics measurements were averaged per genotype. Data was matched by genotype.
